# Supplementary material for: Cell-based high-content approach for SARS-CoV-2 neutralization identifies unique monoclonal antibodies and PI3K pathway inhibitors
Source: Front Cell Dev Biol. 2025 May 22;13:1538934. doi: 10.3389/fcell.2025.1538934 (PMC12142043; doi:10.3389/fcell.2025.1538934)
Supplement: Supplementary file 1 [file DataSheet1.pdf]

Supporting Information

Cell-based high-content approach for SARS-CoV-2 neutralization identifies unique monoclonal antibodies and PI3K pathway inhibitors.

Authors: Carly R. Cabel[1,2], Briana A. Guzman[1], Elaheh Alizadeh[1,2,3], Shuaizhi Li[5], Cameron Holberg[6], Chonlarat Wichaidit[1], Darren A. Cusanovich[1,2,4], Andrew L. Paek[2,7], Gregory R. J. Thatcher[6], Koenraad Van Doorslaer[1,5], Rachel S. Nargi[8], Rachel E. Sutton[8], Naveenchandra Suryadevara[8], James E. Crowe Jr.[8,9,10], Robert H. Carnahan[8,10], Samuel K. Campos[2,5], Curtis A. Thorne[1,2]*

Affiliations:

[1]Department of Cellular and Molecular Medicine, University of Arizona, Tucson, AZ, 85724 USA.

[2]Cancer Biology Graduate Interdisciplinary Program, The University of Arizona, Tucson, AZ, 85724, USA.

[3] The Jackson Laboratory for Genomic Medicine, Farmington, CT, 06032, USA.

[4]Asthma and Airway Disease Research Center, The University of Arizona, Tucson, AZ, 85724, USA.

[5]Department of Immunobiology, BIO5 Institute, University of Arizona, Tucson, AZ, 85724, USA.

[6] Department of Pharmacology & Toxicology, R. Ken Coit College of Pharmacy, University of Arizona, Tucson, AZ, 85721, United States

[7]Department of Molecular and Cellular Biology, The University of Arizona, Tucson, AZ, 85724, USA.

[8]Vanderbilt Vaccine Center, Vanderbilt University Medical Center, Nashville, TN, 85724 USA.

[9]Department of Pathology, Microbiology, and Immunology, Vanderbilt University Medical Center, Nashville, TN, 37232, USA

[10]Department of Pediatrics, Vanderbilt University Medical Center, Nashville, TN, 37232, USA.

***Corresponding author:** Email: curtisthorne@arizona.edu

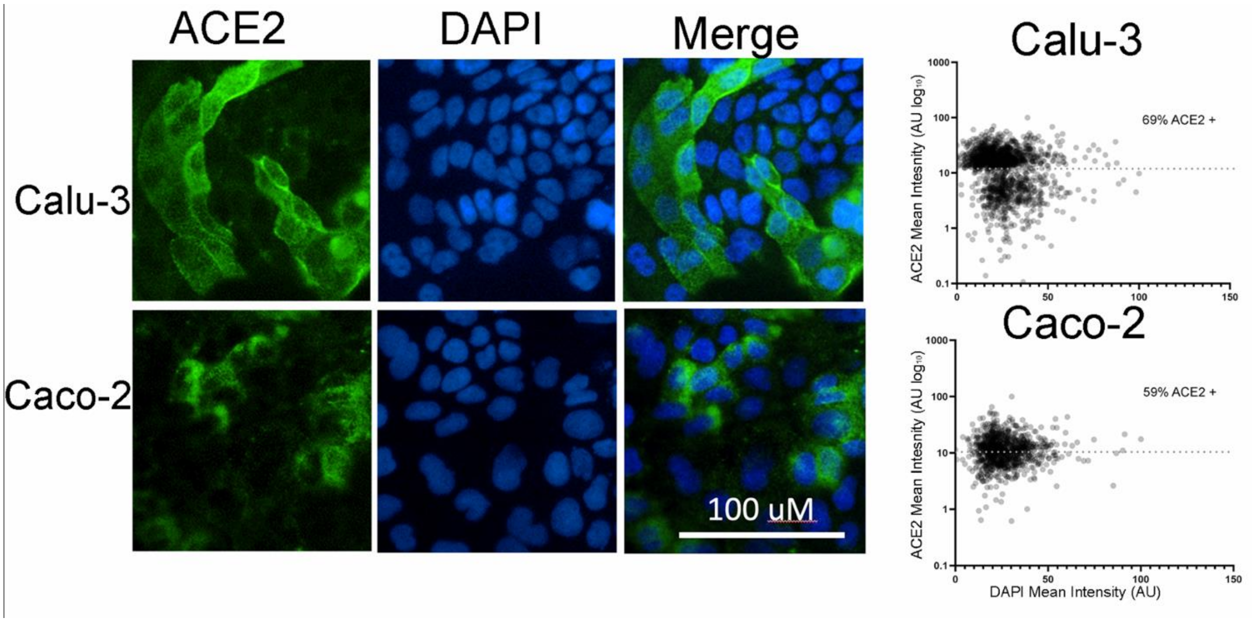

Fig S1: Calu-3 Cells more Permissible to Infection than Caco-2 Cells
Immunofluorescent staining for ACE2 (green), DNA (blue) shows ACE2 receptor expression in Calu-3 and Caco-2 cells. Expression of ACE2 receptor was normalized by ACE2 mean intensity and plotted for Calu-3 cells and Caco-2 cells shown in scatter plot. Percent ACE2 positive cells for each group were calculated based on amount of ACE2 positive cells among all cells in dataset.

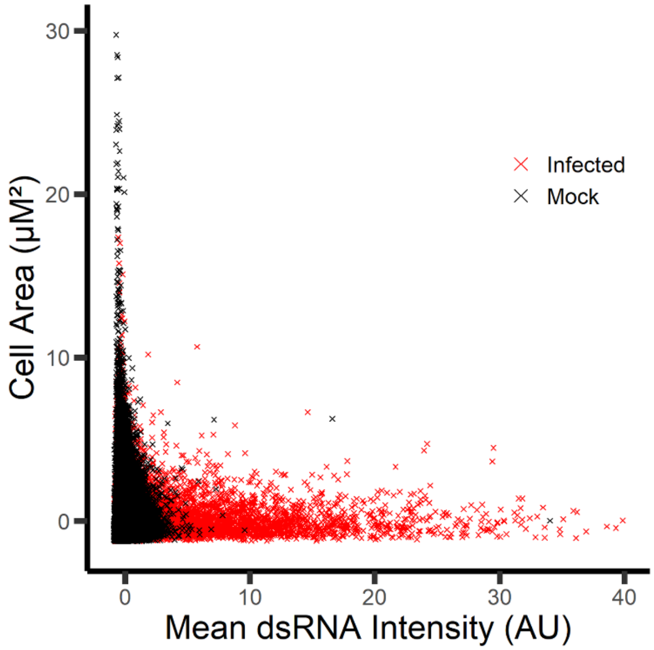

Fig S2: Infection Rate of Calu-3 Cells with SARS-CoV-2
Scatter plot of Calu-3 cells mock or SARS-CoV-2 infected. Cells were stained for dsRNA, imaged, segmented in single-cell objects, and dsRNA quantified.

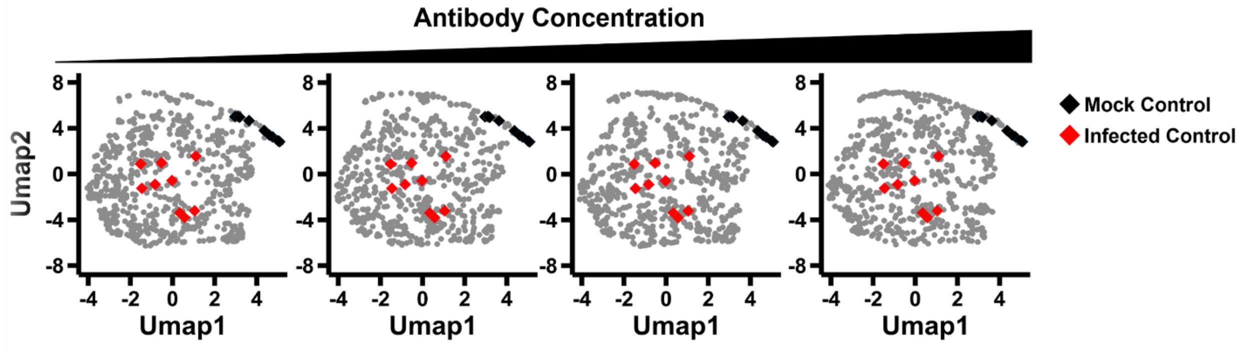

Fig S3: Antibody Response by Concentration

UMAP of antibodies separated by concentration. Right to left show serial dilutions. The shift of the density of the points indicates that with an increasing concentration of antibodies, the cell phenotype subtly moves closer to the mock control (black diamonds) and away from the infected control (red diamonds).

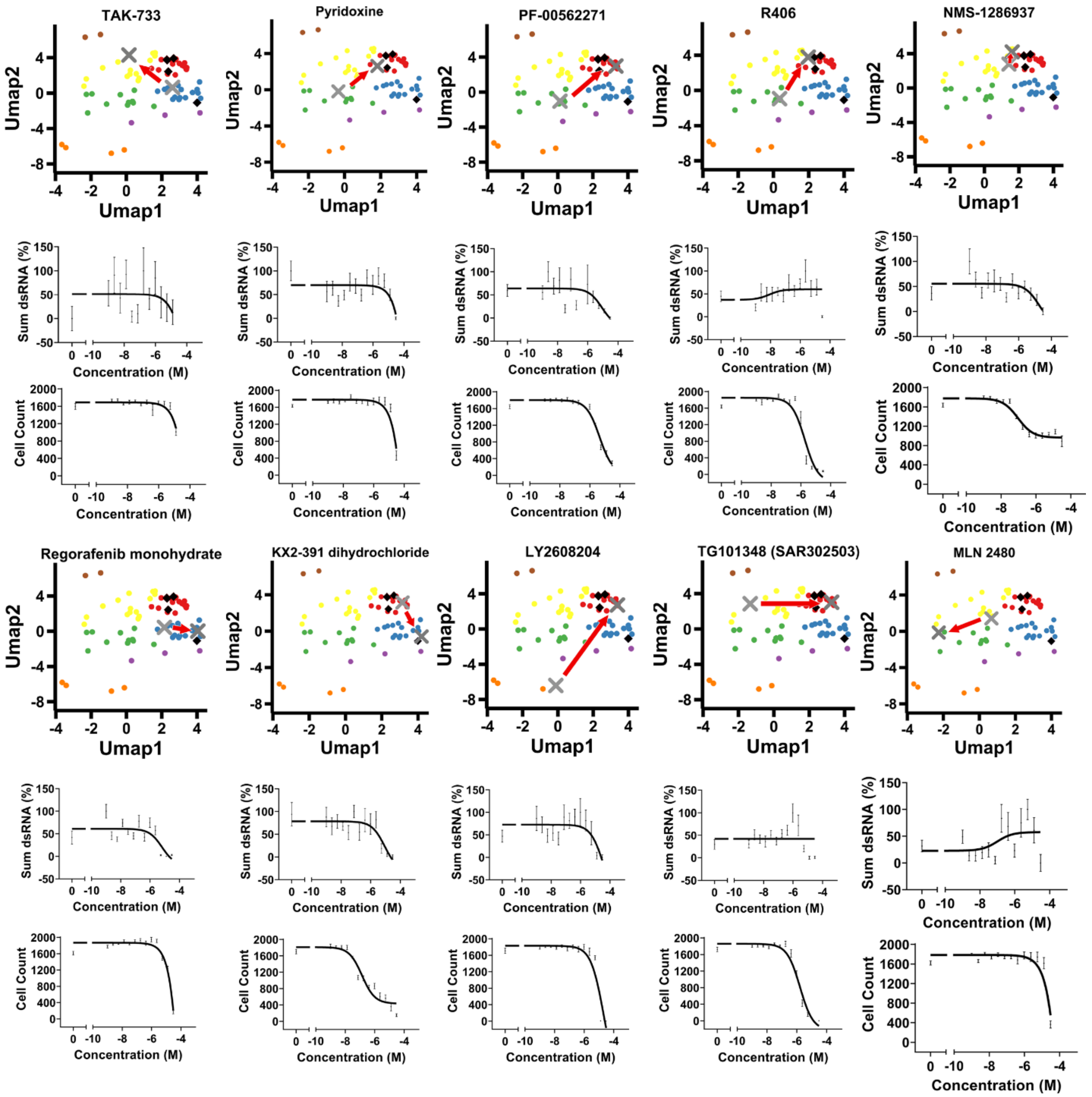
TAK-733
Pyridoxine
PF-00562271
R406
NMS-1286937
Regorafenib monohydrate
KX2-391 dihydrochloride
LY2608204
TG101348 (SAR302503)
MLN 2480
Umap1
Umap2
Sum dsRNA (%)
Cell Count
Concentration (M)

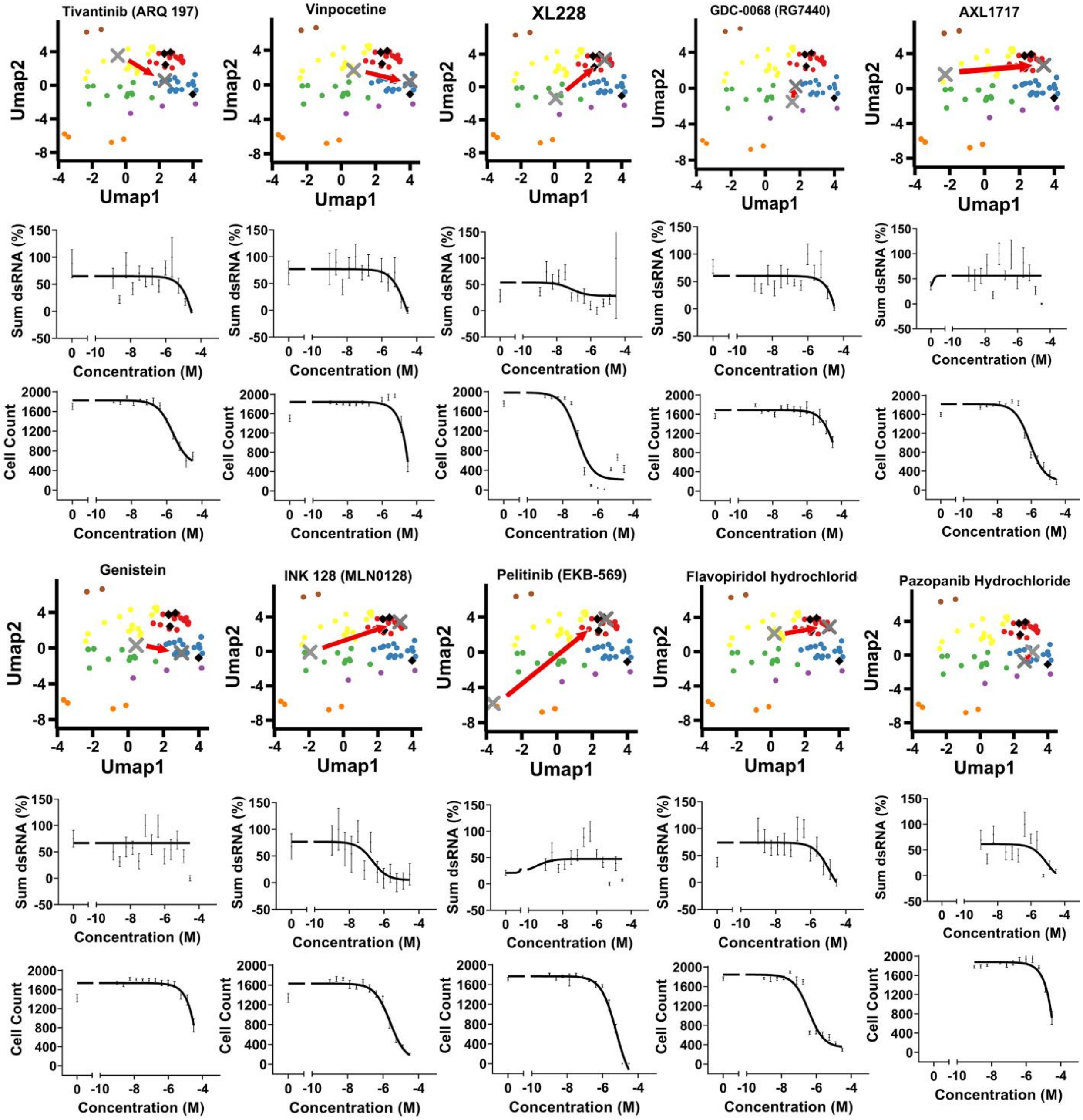
Tivantinib (ARQ 197)
Vinpocetine
XL228
GDC-0068 (RG7440)
AXL1717
Genistein
INK 128 (MLN0128)
Pelitinib (EKB-569)
Flavopiridol hydrochlorid
Pazopanib Hydrochloride
Umap1
Umap2
Sum dsRNA (%)
Cell Count
Concentration (M)

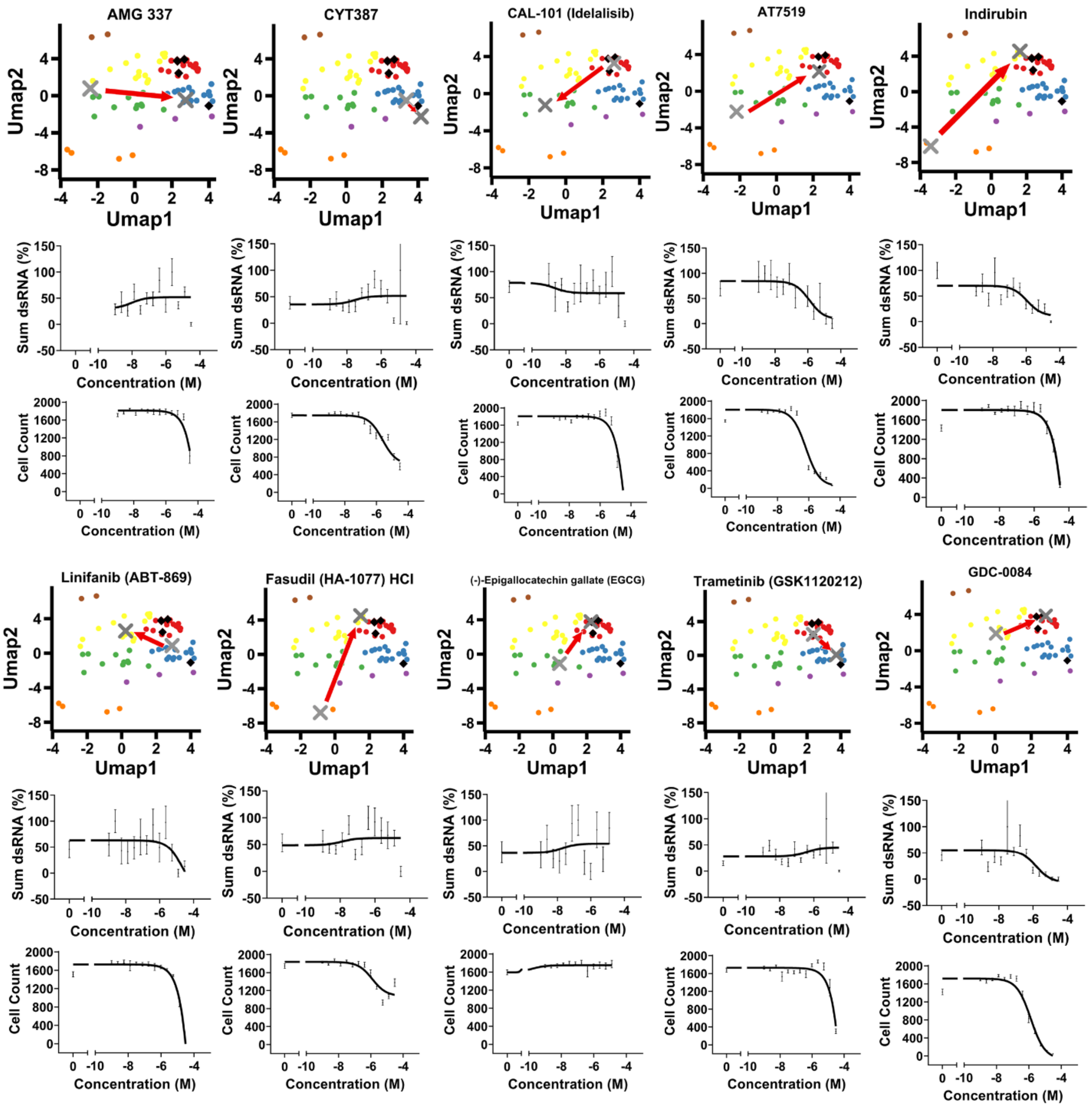

Fig S4: Kinase Inhibitor Screen Results

As in main Figure 5 C-E, a continuation of plots representing the retesting of strong hits from the initial kinase inhibitor screen. The gray X's indicate the low and high doses from the initial screen. The red arrow points from the low dose to the high dose to emphasize the shift in location UMAP space. Sum dsRNA was normalized to percent of response to control. Cell count from dose-response curve was also measured by counting DAPI. Error bars represent SEM.

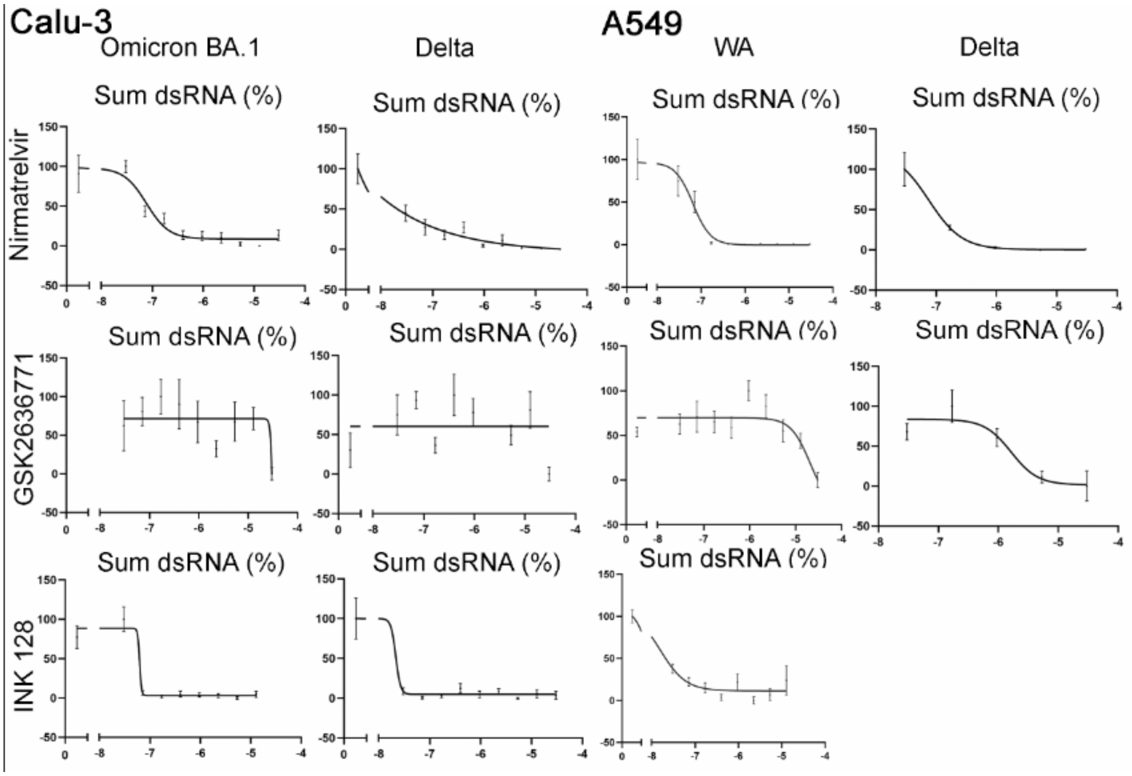

Fig S5: Kinase Inhibitor Screen Results with Omicron Ba.1 and Delta variant in Calu3 and A549 cells

To enhance the robustness and applicability of our findings, we validated the efficacy of our positive control and two compounds from our kinase inhibitor screen across an additional cell line and against two more virus variants, Omicron BA.1 and Delta. The three compounds were tested in an 8-concentration dose response. Sum dsRNA was normalized to percent of response to control. Error bars represent SEM.

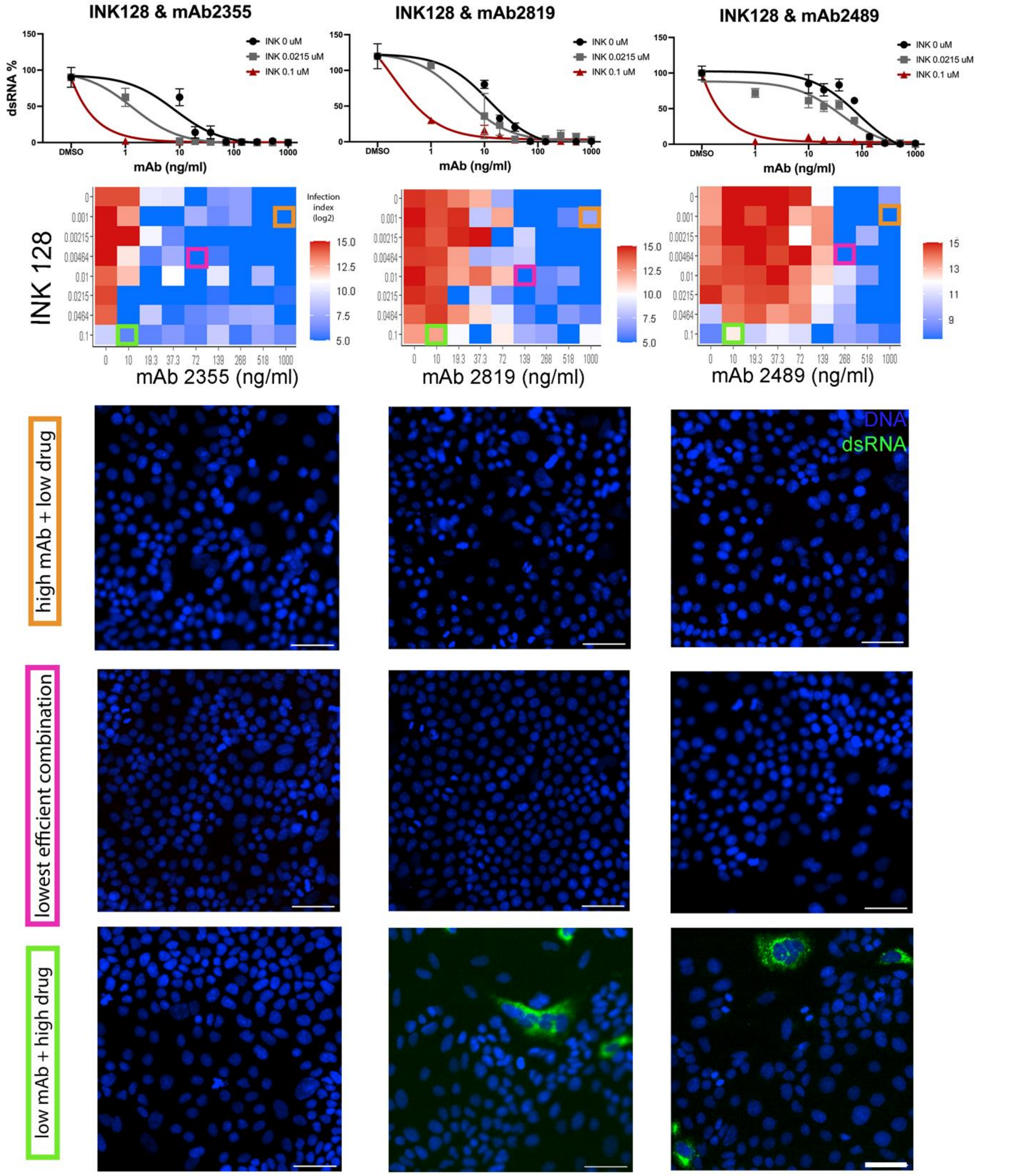

Fig S6: Combination of Neutralizing Antibody and Kinase Inhibitor Results. As in main Figure 6 C-G, a continuation of dose curves, heatmaps, and respective representative images are shown for the combination treatment of monoclonal antibodies 2355, 2819, and 2489, with mTOR inhibitor, INK 128. Representative images of a synergy experiment. The infection index was calculated by measuring the average sum intensity of dsRNA per cell count. DAPI is shown in blue, and dsRNA as viral infection is shown in green. Scale bar = 50µM.

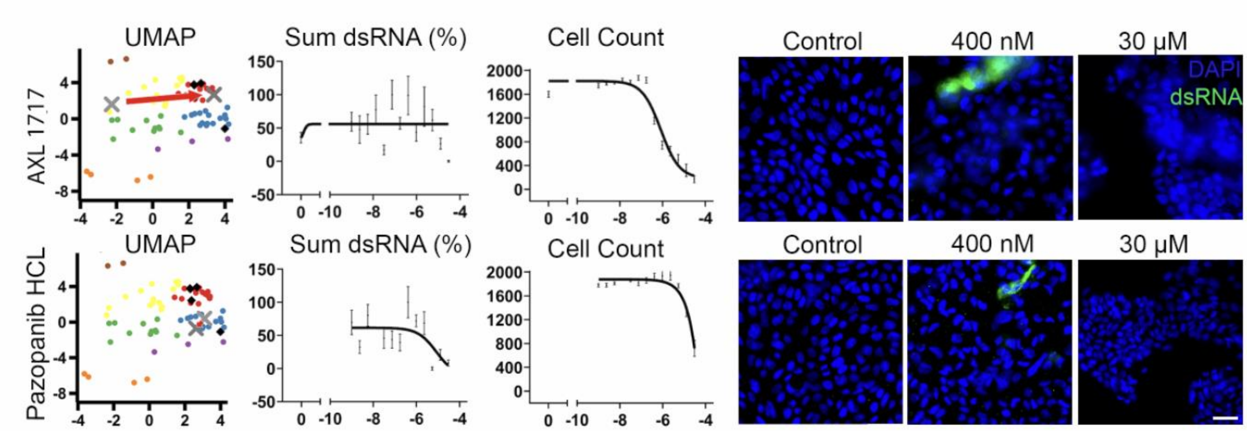

Fig S7: Candidates among our Top Kinase Inhibitor

As in main Figure 5 C-F, a continuation of our candidates from the kinase inhibitor screen are shown. AXL 1717 and Pazopanib HCL were retested in a 12-concentration dose-response curve. The gray X indicates the low and high doses from the initial screen. The red arrow points from the low dose to the high dose to emphasize the shift in location UMAP space. Sum dsRNA was normalized to the percent of response compared to control. Cell count from dose response curve was measured by counting DAPI-stained cells. Right side, representative images of the specified kinase inhibitor with low and high concentrations. Nuclei are stained with DAPI in blue and dsRNA is stained in green. Scale bar = 50μM. Error bars represent SEM.

Table S1: Antibody dsRNA Area Under the Curve (AUC)

	Decreasing Dosage 1:20				
Antibody ID	Dose 1	Dose 2	Dose 3	Dose 4	Sum
2355	0	0	0	0	0
2381	0	0	0	0	0
2504	0	0	0	0	0
2691	0	0	0	0	0
2819	0	0	0	0	0
2832	0	0	0	0	0
2498	0	0	0	0.034282	0.034282
2391	0	0	0	0.042931	0.042931
2499	0.004381	0	0	0.086103	0.090484
2941	0.001553	0.001655	0.001602	0.105511	0.110322
2955	0	0	0.092276	0.02758	0.119856
2308	0	0.006481	0.003528	0.111864	0.121874
2539	0.009391	0	0.032509	0.091817	0.133716
2514	0	0.001193	0.001901	0.146693	0.149787
2531	0.002623	0.002023	0.036593	0.13553	0.176769
2353	0	0	0.094756	0.104947	0.199703
2733	0	0	0	0.25545	0.25545
2290	0	0	0.076816	0.219281	0.296097
2838	0.01561	0.125523	0.093655	0.067743	0.30253
2413	0	0.003581	0.176016	0.126839	0.306435
2859	0.064683	0.080574	0.074623	0.119486	0.339366
2485	0	0.024703	0.011633	0.312655	0.34899
2389	0.028526	0.03034	0.249269	0.141058	0.449193
2813	0.006993	0.004478	0.149629	0.301735	0.462835
2841	0	0	0.122719	0.345178	0.467897
2807	0.006037	0.306831	0	0.175823	0.488692
2341	0.233236	0.086005	0.124472	0.117795	0.561509
2919	0	0.100397	0.250101	0.228387	0.578884
2415	0.055398	0.176151	0.344762	0.010173	0.586484
2952	0	0.049558	0.002553	0.582294	0.634405
2489	0.001251	0	0.042514	0.634815	0.67858
2448	0.121064	0.131853	0.231625	0.202726	0.687268
2417	0.017218	0.218993	0.361723	0.096315	0.694249
2601	0.145502	0.174933	0.129349	0.261008	0.710794
2393	0.171955	0.151889	0.285624	0.1041	0.713567
2406	0.404072	0.098853	0.212046	0	0.71497
2835	0	0.01373	0.05808	0.70013	0.77194
2822	0.418079	0	0.010095	0.371416	0.79959
2589	0.007277	0.060538	0.451686	0.307908	0.827408

2717	0	0.087156	0.502349	0.261643	0.851147
2452	0.194675	0.292466	0.047501	0.323623	0.858266
2625	0.530779	0.109737	0.118047	0.107608	0.866171
2618	0.014705	0.153277	0.635626	0.07575	0.879358
2861	0.446715	0.21932	0.239473	0	0.905509
2399	0.03733	0.543622	0.282536	0.054105	0.917593
2358	0.097848	0.356754	0.455184	0.017341	0.927128
2364	0.002038	0.336557	0.355746	0.264111	0.958451
2398	0	0.072788	0.430367	0.45979	0.962946
2791	0.114229	0.00392	0.453327	0.396017	0.967492
2557	0.082252	0.358082	0.297132	0.232357	0.969823
2344	0.295984	0.404711	0.157044	0.112904	0.970643
2678	0	0	0.990999	0	0.990999
2394	0.212002	0.329673	0.051135	0.460715	1.053525
2369	0.087283	0.374468	0.46364	0.138245	1.063636
2354	0	0	0.807096	0.25728	1.064376
2894	0.007649	0.238954	0.516522	0.307708	1.070834
2295	0.492476	0.213316	0.257153	0.142491	1.105436
2526	0	0.926408	0.107009	0.074027	1.107444
2352	0.029007	0.367883	0.394524	0.323376	1.114791
2946	0.237466	0.154596	0.462878	0.264033	1.118973
2343	0.287072	0.386099	0.288903	0.173125	1.1352
2338	0	0.548486	0.149338	0.449417	1.14724
2572	0.101825	0.082369	0.271511	0.701709	1.157415
2837	0.247833	0.167945	0.365666	0.378159	1.159603
2711	0.958049	0.017952	0	0.204777	1.180777
2374	0.899838	0.01141	0.027563	0.246275	1.185086
2403	0.119641	0.425173	0.460479	0.184419	1.189713
2382	0.018432	0.111559	0.660831	0.415412	1.206234
2573	0.581382	0.055242	0.249787	0.320385	1.206796
2357	0	0.380225	0.50404	0.332963	1.217228
2412	0.146403	0.299915	0.714624	0.058196	1.219138
2386	0.329172	0.299022	0.175305	0.41617	1.219669
2694	0.026897	0.001634	0.360749	0.860283	1.249565
2337	0.251625	0.380675	0.076447	0.559461	1.268209
2350	0.068943	0.922148	0.281516	0	1.272607
2385	0.261145	0.373418	0.525057	0.113881	1.273501
2562	0	0	0.672187	0.604805	1.276992
2397	0.240649	0.147788	0.758177	0.132513	1.279128
2848	0.124972	0.110146	0.371466	0.702326	1.308911
2376	0.234343	0.221284	0.346777	0.511051	1.313454
2414	0.672089	0.430733	0.199986	0.01592	1.318727
2410	0.37644	0.379801	0.141918	0.426466	1.324625
2554	0	0.51631	0.61478	0.22351	1.3546

2447	0.450343	0.229324	0.275503	0.405192	1.360362
2395	0.150956	0.145448	0.422535	0.651117	1.370056
2342	0.009955	0.332174	0.343221	0.68721	1.372559
2379	0.091224	0.39386	0.219579	0.669286	1.373949
2834	0.052781	0.142245	1.158619	0.037858	1.391502
2468	0.31914	0.103657	0.324739	0.648859	1.396395
2535	0.356388	0.345201	0.158675	0.541531	1.401795
2416	0.218385	0.421309	0.329555	0.446087	1.415336
2882	0.017052	0.33713	0.421557	0.647834	1.423573
2846	0.51768	0.277875	0.272542	0.358884	1.42698
2828	0	0.026425	0.193597	1.22975	1.449773
2563	0.01307	0.813588	0.352049	0.272319	1.451026
2543	0.125623	0.570078	0.305744	0.480567	1.482012
2684	0	0.027748	0.160733	1.294534	1.483015
2335	0.58079	0.814525	0.029903	0.062586	1.487804
2937	0.536761	0.555386	0.286201	0.10965	1.487997
2346	0.669053	0.312915	0.48033	0.036328	1.498626
2862	0.006401	0.629049	0.507815	0.355599	1.498865
2375	0.186451	0.074613	0.684057	0.558039	1.50316
2704	0	1.272649	0	0.244495	1.517144
2493	0.530514	0	0.335125	0.653378	1.519016
2392	0.276619	0.21324	0.217442	0.819458	1.526758
2256	0	0.058808	1.34572	0.139548	1.544076
2863	0	0.460135	0.219564	0.86586	1.54556
2367	0.04517	0.438841	0.372166	0.699181	1.555358
2878	0	0.099192	0.419552	1.043154	1.561897
2506	0.166971	0.618363	0.19734	0.589126	1.5718
2935	0.214115	0.805124	0.340223	0.214049	1.57351
2360	0.052582	0.113324	0.850829	0.560743	1.577478
2926	0.252811	0.468572	0.401464	0.45535	1.578197
2461	0	0.635785	0.317831	0.652133	1.605749
2511	0.516783	0.413334	0.582638	0.093859	1.606615
2939	0.114109	0.000752	0.762617	0.733193	1.61067
2564	0.101904	0.626477	0.817961	0.065657	1.611998
2815	0.116341	0.51552	0.860618	0.127089	1.619568
2361	0.184217	0.964469	0.027533	0.448931	1.625149
2333	0.35487	0.060482	0.777561	0.43648	1.629393
2459	0.154364	0.844207	0.146114	0.491426	1.636112
2318	0	0.052203	0.724615	0.879586	1.656403
2460	0.70769	0.131317	0.156018	0.661543	1.656567
2821	0.289918	0.024661	0.010205	1.33734	1.662123
2592	0.465699	0.223065	0.4457	0.527997	1.662461
2432	0.109403	0.523232	0.522051	0.527254	1.681939
2574	0.799457	0.218088	0.49177	0.176032	1.685348

2916	0.907832	0.141205	0.072431	0.576383	1.69785
2703	0.24832	0.706552	0.002597	0.756944	1.714412
2540	0.814356	0.554917	0.101487	0.243749	1.714509
2693	0	0	0.327079	1.388966	1.716045
2400	0.376445	0.332316	0.873278	0.13673	1.718769
2345	0.196692	0.430931	0.080495	1.015436	1.723554
2430	0.305132	0.886057	0.307998	0.230029	1.729216
2561	0.855037	0.191069	0.030162	0.654911	1.731179
2252	0.615725	0.452421	0.0942	0.577859	1.740206
2559	0.709994	0.22225	0.563307	0.249944	1.745496
2409	0.204743	0.140997	1.07789	0.33007	1.753699
2953	0.015388	0.682215	0.468093	0.588481	1.754177
2753	0	0.239413	0.957162	0.559319	1.755894
2437	0	1.27041	0.08044	0.422877	1.773727
2829	0.480649	0.351415	0.179437	0.762956	1.774458
2443	0.30934	0.778197	0.550969	0.137304	1.77581
2914	0.43818	0.19857	0.8892	0.251355	1.777305
2843	0.655609	0.273933	0.23407	0.622801	1.786412
2396	0.857661	0.245742	0.171685	0.511681	1.786769
2585	0.713776	0.511482	0.154945	0.443632	1.823835
2790	0	1.033334	0.498794	0.296622	1.82875
2555	0.341677	0.039223	1.103932	0.347288	1.83212
2875	0.583443	0.522881	0.202138	0.529446	1.837908
2510	0.007015	0.13658	0.552229	1.147378	1.843202
2934	0.163693	0.501926	0.909343	0.268961	1.843923
2626	0.72358	0.150528	0.297478	0.679138	1.850724
2545	0.179466	0.500915	0.406062	0.767213	1.853657
2594	0.608514	0.552958	0.590923	0.101326	1.853722
2404	0.23117	0.254458	0.984758	0.400043	1.870429
2581	0.545522	0.19027	0.595865	0.551178	1.882835
2347	0.724948	0.682072	0.228308	0.252987	1.888315
2958	0.64568	0.354174	0.552877	0.347838	1.90057
2571	0.461495	0.724186	0.427267	0.293574	1.906522
2368	0.253972	0.442238	0.613342	0.600369	1.909922
2362	0.141751	0.191202	1.314281	0.267706	1.91494
2446	0.089231	0.406585	1.201906	0.223678	1.9214
2947	0.275542	0.246366	0.855931	0.566514	1.944353
2455	1.002766	0.199828	0.434014	0.308346	1.944953
2349	0.060691	0.141604	0.630429	1.115654	1.948378
2930	0.641589	0.858584	0.32235	0.138765	1.961288
2556	0.793295	0.229305	0.29786	0.648731	1.969191
2378	0.093424	0.166318	0.337344	1.377973	1.975058
2542	0.404767	0.314378	0.79768	0.459818	1.976643
2548	0.513859	0.977934	0.294219	0.201572	1.987584

2366	0.531135	0.400598	0.302866	0.755505	1.990104
2811	0.423536	0.396974	0.895085	0.277388	1.992982
2532	1.014589	0.31282	0.41259	0.256114	1.996114
2552	0	0	0.810497	1.190243	2.00074
2336	1.650896	0.266734	0.047906	0.036095	2.00163
2454	1.129453	0.090546	0.184607	0.60594	2.010547
2940	0.426445	0.305464	0.927882	0.350883	2.010675
2533	0.37706	0.835602	0.289547	0.512159	2.014369
2428	0.632624	0.627921	0.206235	0.557747	2.024527
2613	0.695691	0.169189	0.799704	0.36979	2.034374
2852	0.657774	0.297805	0.503639	0.584239	2.043458
2466	0.378687	0.580441	0.440047	0.645259	2.044435
2438	0.154442	1.179243	0.54343	0.170128	2.047243
2401	0.156811	0.521486	0.402371	0.968294	2.048962
2929	0.414178	0.306775	0.194403	1.149874	2.06523
2657	1.191936	0.275867	0.4758	0.126874	2.070477
2473	0.146529	0.604353	1.178792	0.150669	2.080342
2418	0.056198	0.378199	1.429597	0.238496	2.10249
2569	0.989304	0.679131	0.074949	0.359415	2.102798
2809	0.318687	0.637997	0.037413	1.114533	2.108629
2348	0.113842	0	0.888421	1.115462	2.117726
2407	1.134749	0.171109	0.327006	0.493035	2.125899
2575	0.445633	1.134548	0.231959	0.324384	2.136525
2673	1.294697	0.374048	0.442164	0.037547	2.148457
2373	0.213239	0.560811	0.4459	0.938604	2.158554
2760	0	0.007486	0.28581	1.874098	2.167394
2356	0.442085	1.104363	0.484089	0.142496	2.173033
2839	0.938884	0.516431	0.367301	0.355801	2.178418
2271	0.21173	0.16063	0.647616	1.159272	2.179247
2634	0.374961	0.21206	0.863673	0.732825	2.183518
2659	1.589007	0.112003	0.28465	0.200268	2.185927
2465	0	0.508297	0.502608	1.177177	2.188082
2844	0.1014	1.157821	0.280763	0.659736	2.19972
2567	1.115589	0.354406	0.49228	0.253124	2.215398
2636	0.155052	0.370799	1.44114	0.248705	2.215697
2688	0	1.583723	0.130143	0.504912	2.218779
2365	0.085222	1.097785	0.234275	0.810347	2.227629
2363	0.114985	0.095152	0.278719	1.742738	2.231594
2604	0.350221	0.472738	0.780117	0.629754	2.23283
2537	0.27045	0.806736	0.695873	0.462369	2.235429
2462	0	0.423711	1.149061	0.684294	2.257066
2440	0.254189	0.050523	0.900349	1.070552	2.275613
2560	1.018998	0.332996	0.492469	0.437034	2.281497
2339	0.106376	1.233398	0.672646	0.273165	2.285586

2384	1.035207	0.121201	1.143034	0.001458	2.300899
2501	0.451894	1.008979	0.359819	0.480725	2.301417
2482	0.298341	0.766363	0.013247	1.23976	2.317711
2696	0.211167	1.068443	0.703583	0.335649	2.318843
2483	0.428655	1.560459	0.20151	0.145682	2.336306
2896	0.492354	0.530429	1.310709	0.006293	2.339785
2771	0.867158	0.529031	0.685605	0.261224	2.343019
2370	0.098117	0.145154	0.096594	2.02104	2.360906
2377	0.474877	0.242109	0.58786	1.059532	2.364379
2411	1.327431	0.070415	0.132722	0.841457	2.372025
2758	0	0.103382	2.102193	0.166927	2.372502
2922	1.614951	0.336082	0.041503	0.381194	2.373728
2402	0.951545	1.031152	0.283216	0.107936	2.373849
2305	0.75736	1.077195	0.46943	0.077795	2.38178
2497	0.34964	1.129116	0.477866	0.428751	2.385373
2913	0.815376	0.606447	0.287847	0.678492	2.388162
2390	0.585182	0.264576	0.606091	0.938199	2.394048
2250	0.093337	0.287196	1.240972	0.773311	2.394815
2429	0.132733	0.520074	0.999661	0.752309	2.404778
2475	0.179415	0.615974	1.253444	0.369167	2.418
2388	0.174657	0.343442	0.568968	1.337097	2.424163
2549	0.619095	0.099582	0.538514	1.183137	2.440328
2541	0.049464	0.467308	0.240273	1.687707	2.444752
2578	0.176606	1.564527	0.434505	0.276941	2.452579
2949	0.444744	0.373231	0.483852	1.1524	2.454227
2380	0.221627	0.754538	1.350433	0.140705	2.467303
2960	1.534363	0.114576	0.192588	0.643465	2.484992
2690	0.622979	0.046269	1.339104	0.495211	2.503563
2327	0.1328	0.590456	0.41555	1.365634	2.50444
2576	0.682481	0.742829	0.25657	0.823402	2.505282
2491	0.174392	0.460697	0.738834	1.133394	2.507316
2570	0.140467	1.07467	0.687502	0.608878	2.511517
2775	1.299123	0.687567	0.140192	0.385084	2.511967
2579	0.265231	0.965814	0.833333	0.473383	2.537761
2709	0	0.109951	1.38866	1.04177	2.540381
2903	1.096105	0.493658	0.75144	0.204226	2.545429
2450	0.214813	0.787595	1.384206	0.170969	2.557584
2851	0.208484	0.167481	0.430503	1.753556	2.560025
2383	0.048924	0.288955	2.007346	0.237829	2.583055
2371	0.534277	0.762651	0.892764	0.404278	2.59397
2759	0	0	1.965544	0.63033	2.595874
2817	0.005356	0.357722	0.140907	2.093877	2.597862
2442	1.083055	0.13546	1.174898	0.205286	2.598699
2823	0.003272	0.516486	1.736826	0.34433	2.600914

2876	1.720107	0.109929	0.477403	0.305643	2.613081
2631	1.325918	0.127146	0.742973	0.419693	2.615731
2853	0.421391	1.157036	0.958727	0.079266	2.61642
2484	0.507149	0.825035	0.5413	0.751667	2.62515
2472	0.345571	1.862283	0.121787	0.301294	2.630935
2936	0.221946	0.265064	0.638636	1.508461	2.634108
2606	0.87735	0.174413	0.676067	0.90816	2.635991
2519	0.708668	0.154404	1.488684	0.289525	2.641281
2632	0.424483	2.121128	0.004986	0.117913	2.668509
2826	0.671259	0.249867	1.445428	0.323406	2.68996
2959	0.32313	0.826346	1.338576	0.204437	2.692488
2708	0.434806	0.067032	1.264647	0.940318	2.706803
2477	0.69976	1.021806	0.579727	0.416099	2.717392
2854	0.908759	0.462629	1.309145	0.039986	2.720519
2524	0.697453	1.176794	0.440475	0.412709	2.72743
2612	0	0.554534	0.224019	1.949856	2.72841
2910	0.724572	0.42936	0.096231	1.4818	2.731963
2340	0.405881	0.608642	1.01904	0.699204	2.732767
2593	0.686376	1.081858	0.229865	0.739951	2.73805
2595	0.679911	0.937294	0.932758	0.21951	2.769474
2591	0.402885	1.205658	0.276198	0.889548	2.774289
2879	0.273402	1.085358	0.994306	0.424909	2.777975
2692	0.902044	0.25634	0.505862	1.123678	2.787924
2599	1.009643	1.324581	0.190151	0.276065	2.800441
2294	1.934737	0.429833	0.184398	0.255376	2.804343
2685	0.139117	0.511074	1.324108	0.84292	2.817219
2766	1.092352	0.919182	0.506329	0.316688	2.83455
2590	1.400176	0.049221	1.113328	0.287011	2.849735
2503	0.264596	0.42048	2.168865	0.012424	2.866365
2951	0.608249	1.179258	0.323681	0.776196	2.887384
2768	0	0.003208	2.445999	0.441941	2.891149
2433	1.241332	0.563021	0.572596	0.514215	2.891164
2885	0.894135	0.696733	0.993438	0.307704	2.89201
2300	0	1.164009	1.628118	0.10412	2.896247
2658	0.712881	0.738007	0.978406	0.474859	2.904153
2420	0.935688	0.858583	0.832184	0.279573	2.906028
2800	0.602346	0	1.185292	1.123653	2.91129
2359	0.982892	0.157723	0.734579	1.044334	2.919529
2812	0	0.652157	1.661421	0.619108	2.932686
2906	0.334544	0.188858	1.736906	0.673633	2.933941
2587	0.106294	0.090861	0.592553	2.144548	2.934256
2492	0.529449	1.360578	0.590641	0.453655	2.934322
2534	1.081769	0.795714	0.417526	0.645166	2.940176
2860	0.154511	0.427595	1.795815	0.570522	2.948443

2950	1.258118	0.282895	0.44058	0.980222	2.961815
2507	1.692323	0.48419	0.270763	0.538269	2.985546
2880	0.432286	2.088868	0.449987	0.027319	2.99846
2833	2.476407	0.200458	0.073346	0.259536	3.009747
2605	0.425602	0.576104	1.083128	0.935922	3.020756
2730	1.044371	0.480054	1.027419	0.478962	3.030806
2810	0.327821	0.046971	0.205921	2.452597	3.03331
2516	0.698551	0.474628	1.187512	0.684446	3.045138
2874	0.646383	0.06213	1.724719	0.612797	3.046029
2270	0.11752	1.190269	0.743102	0.999628	3.050519
2330	2.559777	0.006345	0.002877	0.488643	3.057642
2640	0.976442	1.268751	0.216357	0.609949	3.071499
2836	0.666574	0	2.143293	0.262242	3.072108
2897	0.818057	0.272647	1.4404	0.555496	3.086601
2773	0.616996	0.159014	0.710161	1.611808	3.097979
2508	0.483974	2.026065	0.219587	0.373237	3.102863
2820	0.577671	0.142407	0.993802	1.399273	3.113154
2847	1.246954	0.987752	0.302332	0.581503	3.118541
2405	0.850476	0.957375	0.401761	0.910384	3.119997
2495	0.195216	0.264658	1.098372	1.587077	3.145323
2439	0.611149	1.017335	0.577832	0.940167	3.146483
2319	0.384219	2.281761	0.327719	0.163335	3.157035
2739	0.320388	1.121503	0.312989	1.403963	3.158843
2866	0.113986	0.796302	1.301998	0.95649	3.168777
2727	1.874804	0	0.924169	0.372811	3.171784
2471	0.838134	0.315647	1.288737	0.742025	3.184543
2470	0.872472	0.941246	0.650704	0.723046	3.187468
2444	1.861143	0.191064	0.856908	0.292462	3.201577
2321	1.291482	0.265515	1.081455	0.568707	3.207159
2676	0.044427	0	2.576741	0.59741	3.218579
2915	0.566564	0.848076	0.130242	1.685248	3.230131
2943	0.65733	0.696995	1.251581	0.630605	3.23651
2248	0	0.052495	0.784543	2.415381	3.252419
2796	0.372652	0.83848	1.751137	0.292576	3.254846
2253	0.037016	0.398667	1.373734	1.447784	3.257201
2945	0.070404	0.007698	2.623292	0.556652	3.258046
2699	0.714206	0.390624	1.290591	0.872785	3.268206
2449	0.218043	0.500885	1.527406	1.03217	3.278505
2289	0.149203	1.795046	0.397933	0.947427	3.289609
2435	0.885247	0.056613	2.069323	0.289824	3.301007
2312	0.76752	0.338643	0.740425	1.458893	3.305481
2500	1.048573	0.534611	1.075225	0.647977	3.306386
2558	0.038586	1.066154	0.691196	1.516812	3.312748
2881	1.253246	0.014792	1.084875	0.963503	3.316417

2434	0	1.487775	1.658926	0.174679	3.32138
2467	0.272411	1.077583	0.813703	1.159783	3.32348
2858	0.21027	1.739736	0.054125	1.321671	3.325802
2596	1.174151	0.417654	0.580184	1.169655	3.341644
2487	0.29765	0.975431	1.602943	0.469963	3.345987
2608	0.785129	1.180948	0.943813	0.436742	3.346633
2870	1.057279	1.511165	0.467668	0.32461	3.360721
2827	0.943154	1.996329	0.289618	0.143307	3.372409
2600	0.942805	0.492727	0.534917	1.40489	3.37534
2686	0.187591	0.39484	1.768102	1.029765	3.380297
2873	0	0.14291	0.955834	2.285344	3.384088
2954	0.905963	0.109908	1.268037	1.110055	3.393962
2921	1.262414	0.590617	0.534419	1.018866	3.406316
2932	0.850754	1.216013	0.116125	1.22488	3.407773
2784	2.018725	0.274434	0.136008	0.999514	3.42868
2857	1.195941	1.781942	0.33593	0.131662	3.445475
2701	0.83222	0.72931	1.019758	0.872047	3.453336
2804	0.495346	1.609722	0.60951	0.76743	3.482008
2933	0.395051	1.234306	1.376653	0.485877	3.491888
2957	0.883401	1.396456	0.830193	0.385202	3.495252
2740	0.094724	1.340266	0.6718	1.398901	3.505691
2783	0.054238	1.608897	1.130616	0.722014	3.515765
2583	0.724292	1.448492	1.046095	0.300162	3.519042
2240	0.091999	1.002321	1.293335	1.136083	3.523738
2544	0.673099	1.096828	1.511712	0.26762	3.549259
2610	0.574648	0.674287	1.967785	0.353725	3.570445
2527	1.157865	0.662134	0.808842	0.956485	3.585327
2927	1.263178	0.702857	0.95453	0.669286	3.58985
2656	1.056606	1.344103	1.025041	0.174937	3.600687
2547	0.220132	0.389692	1.961369	1.03023	3.601423
2898	0.844347	0.053826	1.775087	0.928985	3.602246
2872	1.871882	0.136868	0.218835	1.376003	3.603589
2311	0.603066	0.990731	1.454258	0.564423	3.612478
2607	2.161859	1.3231	0	0.140323	3.625282
2726	2.690052	0.451049	0.439778	0.050539	3.631418
2478	0.157754	0.872027	1.619303	0.982693	3.631777
2831	0.08072	0.975378	1.172352	1.414764	3.643214
2284	1.261587	0	2.225393	0.160921	3.647901
2891	0.786903	0.040726	0.802059	2.025588	3.655276
2494	2.611778	0.313818	0.592191	0.137518	3.655305
2627	1.768379	0.963499	0.160998	0.771734	3.66461
2868	1.82658	0.789256	0.646195	0.416249	3.67828
2752	0	0	0.044367	3.636947	3.681314
2481	0.618931	1.251558	1.371233	0.44288	3.684603

2924	2.256003	0.166114	0.492025	0.778859	3.693001
2707	1.542038	0.266912	0.355818	1.547242	3.71201
2565	0.673954	0.888825	1.033368	1.117322	3.713469
2887	1.641034	1.420841	0.471717	0.192201	3.725793
2806	1.143784	0.695195	1.595006	0.3124	3.746386
2710	0	0.842823	0.663308	2.271368	3.7775
2902	0.050625	0.961534	0.168742	2.602675	3.783576
2419	1.445223	0.320896	1.910177	0.138585	3.814881
2525	0.440102	1.365071	1.300733	0.723577	3.829483
2808	0.48159	1.421818	0.876285	1.057775	3.837468
2314	0.616153	0.507328	2.046629	0.673812	3.843922
2842	0.763533	1.429609	0.841182	0.819686	3.854011
2705	0.489019	2.461339	0.394579	0.510697	3.855633
2445	0.009569	0.779632	2.71394	0.352501	3.855641
2272	1.497868	1.610787	0.015756	0.735152	3.859563
2580	2.034218	0.577762	0.309242	0.938746	3.859967
2588	1.742046	1.118902	0.76726	0.234344	3.862552
2288	0.8046	0.770073	1.9931	0.304209	3.871982
2886	0.704895	0.599633	0.636703	1.938564	3.879794
2956	0.38216	2.080602	0.579427	0.856951	3.899139
2474	0.759953	1.625318	1.044963	0.471408	3.901642
2463	0.265101	1.397866	0.272448	1.9685	3.903916
2719	1.650058	0.314923	1.939047	0	3.904027
2923	0.774479	1.071361	0.179595	1.916124	3.94156
2258	0	0	0.102389	3.843831	3.94622
2637	0.638444	1.252739	1.83853	0.23345	3.963163
2905	1.01936	1.50077	0.133557	1.31256	3.966247
2431	0.46111	0.270341	0.592914	2.654908	3.979274
2456	0.19879	1.612619	1.843137	0.355586	4.010132
2818	0.722297	1.295621	0.498211	1.50787	4.024
2278	0.280655	0.635806	0.269609	2.84357	4.02964
2849	0.787507	0.59506	0.700966	1.958888	4.042421
2551	0.532783	1.546962	0.521424	1.442023	4.043192
2716	0.884627	1.680342	0.583474	0.90732	4.055762
2476	1.11301	1.26864	0.874079	0.804481	4.060209
2855	0.432946	1.118378	1.893954	0.615561	4.060839
2287	0	0	0.383088	3.708513	4.091601
2469	1.474106	0.42491	1.729608	0.467958	4.096583
2695	1.962266	0.001663	0.809537	1.324999	4.098465
2372	0.838697	0.144096	1.985034	1.170071	4.137898
2244	1.745029	1.340812	1.033636	0.022405	4.141882
2765	0.124239	0.154196	1.828453	2.054363	4.161252
2254	0.811647	0.478806	2.83069	0.041641	4.162784
2615	2.263812	0.659327	0.78372	0.458331	4.16519

2255	0.762422	2.173102	1.027659	0.20567	4.168853
2918	1.067748	1.871618	0.045696	1.208353	4.193416
2265	2.057772	1.978356	0.029586	0.185094	4.250808
2715	2.118506	0.493666	0.773498	0.870763	4.256434
2655	0.975216	0.153584	2.039176	1.100827	4.268802
2303	0.381665	2.191874	1.047918	0.649546	4.271004
2263	0.169547	2.71614	0.840633	0.544744	4.271065
2624	0.02572	0.567881	1.642488	2.044789	4.280878
2529	0.930996	1.723346	0.838504	0.80292	4.295766
2512	1.742461	1.356153	0.670285	0.549348	4.318248
2689	1.093448	0.193762	1.945819	1.086871	4.3199
2351	2.336269	0.450563	1.059853	0.500507	4.347192
2865	0.773338	0.270855	1.623719	1.722469	4.390381
2509	0.335843	0.332245	1.273746	2.482522	4.424356
2742	0.457107	0.596538	0.190336	3.181761	4.425742
2877	1.756053	0.917913	0.644969	1.119217	4.438153
2480	2.904961	0.654033	0.610034	0.269399	4.438428
2647	1.462575	0.433643	1.45835	1.094359	4.448927
2893	0.819472	2.34139	0.956589	0.342372	4.459823
2931	2.217377	1.310751	0.317199	0.6273	4.472627
2917	2.471612	0.331661	1.603714	0.081183	4.488171
2611	0.539473	1.713788	1.633555	0.621638	4.508455
2513	0.24859	2.171325	1.347141	0.745331	4.512386
2387	0.36027	0.705018	2.148238	1.299872	4.513397
2538	0.726746	1.037903	2.0023	0.762423	4.529372
2422	2.366065	0.849035	0.346139	0.987479	4.548717
2502	2.350629	0.249116	0.531921	1.43963	4.571296
2546	2.9117	0.158463	1.209431	0.304696	4.584291
2629	0.522781	1.048576	0.968155	2.046107	4.585619
2425	0.268247	2.351296	0.83631	1.144469	4.600322
2436	1.197715	0.57094	2.053194	0.809676	4.631525
2925	1.526677	0.568074	1.896942	0.654464	4.646157
2239	3.451201	0	0.802257	0.399391	4.652849
2275	1.692303	0.28207	1.716051	0.990294	4.680719
2667	1.731128	0.60735	2.317099	0.037809	4.693386
2458	0.261827	0.944515	0.615895	2.886701	4.708938
2900	1.626145	2.135005	0.457512	0.502182	4.720843
2486	1.656098	1.127544	0.436516	1.50801	4.728168
2633	2.386824	0.913598	1.384456	0.047152	4.73203
2845	0.521894	3.823186	0	0.393318	4.738398
2757	1.467957	1.373592	0.403574	1.518895	4.764018
2944	1.072854	0.568563	2.145282	0.999756	4.786455
2550	0.157032	1.961027	0.779399	1.899645	4.797104
2920	1.022593	0.476097	1.735831	1.586067	4.820588

2643	0.137223	1.399201	0.827213	2.46169	4.825326
2644	0.030564	0.795826	2.544496	1.475129	4.846014
2242	1.77706	0.889625	0.25865	1.924448	4.849783
2899	2.639707	0.638841	0.874062	0.716415	4.869025
2938	0.183271	2.817753	0.488971	1.381749	4.871743
2315	0.740436	1.311781	0.640166	2.18088	4.873263
2269	0.612592	1.048393	1.998974	1.233455	4.893414
2259	3.754996	0	0.780828	0.392897	4.928721
2770	1.715946	0.924277	1.696159	0.605572	4.941954
2840	0.264993	2.110674	0.523884	2.077206	4.976756
2749	0	2.028693	0.459038	2.493258	4.980989
2794	1.388363	0.697478	0.388611	2.507996	4.982449
2530	1.916634	1.461464	1.550606	0.059429	4.988132
2668	0.238326	0.117916	0.079217	4.561806	4.997264
2928	2.436036	0.961538	0.158808	1.464944	5.021326
2746	0.48958	1.516368	1.779193	1.252143	5.037285
2722	0	0	0.698992	4.338873	5.037865
2299	0.380178	3.353856	0.626664	0.706286	5.066983
2641	0.124514	2.301753	0.628608	2.024289	5.079164
2249	0.888651	2.002809	0.771103	1.427562	5.090125
2681	0.207108	3.346413	0.763858	0.780788	5.098165
2520	0.232665	1.014881	1.792002	2.061714	5.101262
2262	0	0.00539	4.209373	0.905414	5.120177
2424	2.0185	1.462764	0.768137	0.881945	5.131346
2856	0.548115	0.674569	1.319066	2.590084	5.131834
2298	2.079283	1.994794	0.552587	0.535604	5.162268
2528	0.346927	3.772174	0.515171	0.556095	5.190367
2697	0.452974	1.987003	0.504455	2.29526	5.239692
2683	0.427104	2.158931	1.009699	1.687746	5.28348
2747	0.598363	0.587451	0.596529	3.501375	5.283718
2304	1.312304	1.813893	1.977577	0.221196	5.32497
2427	0.481441	2.074797	1.17487	1.625385	5.356493
2948	2.229177	0.574703	1.738212	0.825806	5.367897
2586	0.733797	1.064951	0.419991	3.152221	5.370959
2408	0.915583	2.786736	1.538323	0.130402	5.371045
2320	2.090958	0.840637	1.211552	1.259935	5.403082
2441	1.20677	1.281155	0.668073	2.248548	5.404546
2316	0.682856	0.537013	1.357986	2.829462	5.407317
2297	1.018575	2.098834	0.836052	1.522868	5.476329
2942	0.484685	1.695262	2.102649	1.218932	5.501529
2761	1.138245	2.268637	0.924992	1.181461	5.513334
2700	0.878394	3.364868	0.520052	0.757242	5.520556
2630	0.846015	2.466707	1.633873	0.614291	5.560887
2687	0.092166	3.33726	0.004467	2.128646	5.562539

	2598	3.865192	0.144248	1.059888	0.512382	5.581711
	2518	0.042155	1.742327	0.956869	2.858207	5.599558
	2521	0	0.015785	1.198975	4.410766	5.625526
	2680	0.818027	3.177143	1.339538	0.361159	5.695867
	2674	1.050458	1.63736	0.917391	2.183942	5.789151
	2268	0.007952	0.475866	3.8697	1.490803	5.844321
	2767	1.618446	0.225889	0.606283	3.416158	5.866776
	2660	0.265079	2.382597	1.163026	2.099268	5.90997
	2675	0.743744	3.24673	1.422245	0.56877	5.98149
	2577	2.356539	2.351835	1.016934	0.280745	6.006053
	2883	1.266302	1.405263	1.235587	2.108566	6.015718
	2257	1.022393	1.445134	3.296532	0.269414	6.033473
	2282	0.562751	1.58388	2.747633	1.188821	6.083086
	2663	3.020849	1.607538	0.605627	0.860131	6.094144
	2553	2.606177	1.563295	0.956079	0.983606	6.109157
	2421	3.87785	0.324365	1.338627	0.570095	6.110938
	2496	0.283336	0.521325	2.858787	2.4498	6.113248
	2280	0.927588	1.746651	1.8458	1.597295	6.117335
	2720	0.293991	3.801045	0.740448	1.317462	6.152947
	2619	0.173953	3.139212	2.171493	0.717953	6.20261
	2802	0.160471	3.580033	1.777425	0.698147	6.216076
	2755	0.971074	2.067905	2.774783	0.41158	6.225342
	2247	3.711992	1.828487	0.362863	0.357193	6.260537
	2731	0.281138	3.128943	1.357563	1.50031	6.267954
	2789	0.257385	2.697508	0.321122	3.106149	6.382164
	2723	0.970348	0.606496	1.234118	3.57454	6.385501
	2661	0.890476	2.681299	1.353883	1.472273	6.397931
	2505	0.535698	2.560604	0.540517	2.767624	6.404444
	2628	0.002478	2.495173	0.106355	3.818809	6.422815
	2329	0.233257	3.964642	0.991486	1.279545	6.468929
	2774	3.611388	1.553196	0.810793	0.497611	6.472988
	2266	0.504572	1.405141	2.388489	2.196952	6.495154
	2664	1.362638	1.346564	0.331328	3.456685	6.497215
	2623	1.385885	0.195175	1.395198	3.573984	6.550243
	2464	0.0466	4.631908	1.34616	0.530571	6.55524
	2522	2.84452	2.204184	0.640346	0.881658	6.570708
	2799	0.418343	2.527779	0.545468	3.169877	6.661466
	2786	0.583413	2.865467	0.332974	2.89946	6.681314
	2620	2.299175	2.107343	1.289763	1.012359	6.70864
	2725	1.411242	0.262982	3.948434	1.096098	6.718756
	2653	2.568896	1.962098	1.967802	0.23894	6.737736
	2734	0	4.205452	1.707796	0.824653	6.737901
	2597	0.663317	4.97502	0.359564	0.778872	6.776772
	2781	3.256189	2.029139	1.18915	0.32298	6.797458

	2451	0.240714	1.311897	2.145793	3.109637	6.808042
	2651	3.069529	0.903097	2.407581	0.437687	6.817894
	2279	1.771889	2.663787	0.452433	1.941068	6.829178
	2622	0.848198	0.17072	4.547634	1.330314	6.896866
	2241	0.03239	2.339776	3.763765	0.761661	6.897591
	2792	0.653998	1.306054	1.961777	3.009888	6.931717
	2614	0.256356	2.074812	2.570895	2.045226	6.947289
	2621	1.224601	0.530474	1.619239	3.575631	6.949945
	2331	0.022986	2.104255	2.726514	2.097743	6.951497
	2286	0.515423	0.543983	5.133594	0.857297	7.050297
	2738	0.760353	0.673647	4.455318	1.2039	7.093217
	2816	3.715713	0.548977	2.330875	0.527887	7.123452
	2801	1.404405	0.520817	2.204376	3.083954	7.213552
	2302	6.666753	0.06403	0.292334	0.216842	7.239959
	2326	3.577067	0.817746	0.744181	2.129881	7.268875
	2798	0.718823	4.254161	2.300924	0.011304	7.285212
	2457	0.827378	0.908466	3.36978	2.191696	7.297321
	2568	4.5916	1.524003	0.302129	0.940016	7.357749
	2679	2.736157	2.577316	0.870391	1.178221	7.362085
	2277	1.204263	0.999815	0	5.205417	7.409495
	2426	0.843298	1.2308	1.748224	3.622909	7.445231
	2698	3.179344	1.069359	0.166531	3.041507	7.456741
	2488	2.038751	3.378169	1.526117	0.560014	7.503051
	2824	1.072917	4.701563	1.347934	0.423209	7.545623
	2515	0.698846	3.689043	1.223621	1.982759	7.594269
	2788	3.075928	1.709109	1.374249	1.489281	7.648567
	2830	0.007692	0.129935	7.281402	0.240213	7.659242
	2825	0.518494	2.341812	3.903186	0.988883	7.752375
	2756	1.643784	0.582845	0.005483	5.522297	7.75441
	2301	0.112513	1.243844	0.081955	6.339895	7.778206
	2737	1.92082	1.23826	3.89568	0.800776	7.855536
	2584	0.834254	4.000905	1.9794	1.071324	7.885883
	2260	1.193882	3.458901	2.273113	0.961102	7.886998
	2267	0.02514	1.312566	1.676944	4.939306	7.953956
	2713	0	0.803841	1.200443	5.952451	7.956736
	2777	0.884296	0.456733	2.155999	4.507551	8.004578
	2273	2.764608	3.14717	1.813427	0.303755	8.028959
	2724	1.372557	4.478695	1.969127	0.295582	8.115961
	2702	1.579163	1.688822	4.490703	0.481347	8.240036
	2603	3.478313	4.216949	0.257065	0.315696	8.268023
	2328	0.145857	6.309081	1.013706	0.809266	8.27791
	2677	0	0	1.68414	6.595049	8.279189
	2423	1.596819	3.756314	2.376673	0.614794	8.3446
	2735	1.070837	0.657341	1.574309	5.060364	8.362852

**2317**	**3.333783**	**2.533509**	**0**	**2.540936**	**8.408229**
**2732**	**3.499855**	**0.201377**	**1.808126**	**2.942067**	**8.451426**
**2638**	**3.593224**	**0.580947**	**1.902549**	**2.388613**	**8.465334**
**2261**	**3.570568**	**2.005172**	**0.194146**	**2.967858**	**8.737743**
**2721**	**1.179135**	**0.509264**	**6.835259**	**0.258237**	**8.781896**
**2743**	**2.039485**	**0.763519**	**5.440141**	**0.560081**	**8.803226**
**2296**	**0.455985**	**2.659943**	**2.034053**	**3.664625**	**8.814605**
**2850**	**1.140381**	**6.446992**	**0.840501**	**0.438067**	**8.86594**
**2283**	**2.938858**	**1.214691**	**2.312287**	**2.408491**	**8.874327**
**2814**	**0.110413**	**0.142368**	**8.31385**	**0.357136**	**8.923766**
**2714**	**5.167299**	**1.691586**	**1.17452**	**1.133729**	**9.167134**
**2274**	**4.238172**	**1.40995**	**1.977982**	**1.657063**	**9.283167**
**2602**	**3.544736**	**0.90509**	**2.030318**	**2.834378**	**9.314521**
**2453**	**0.443999**	**0.304188**	**4.345566**	**4.291268**	**9.38502**
**2706**	**1.944133**	**3.72675**	**3.304427**	**0.533527**	**9.508837**
**2332**	**0.300811**	**7.160398**	**1.435792**	**0.725207**	**9.622209**
**2744**	**6.293804**	**1.643438**	**0.391353**	**1.367935**	**9.69653**
**2490**	**0.1235**	**6.738118**	**0.841717**	**2.021377**	**9.724712**
**2310**	**0.325897**	**4.158082**	**3.280974**	**2.064434**	**9.829386**
**2323**	**5.67428**	**0.289326**	**3.827468**	**0.065618**	**9.856692**
**2616**	**0.355791**	**2.303506**	**2.329101**	**4.924116**	**9.912514**
**2754**	**1.153772**	**2.507235**	**0.755658**	**5.653636**	**10.0703**
**2728**	**0.523353**	**0.774776**	**7.753078**	**1.030164**	**10.08137**
**2639**	**5.777024**	**1.015276**	**2.265184**	**1.045051**	**10.10254**
**2718**	**0.346026**	**6.726148**	**0**	**3.040884**	**10.11306**
**2322**	**1.251465**	**0.846545**	**0.818972**	**7.266298**	**10.18328**
**2246**	**1.789292**	**6.933244**	**0**	**1.550209**	**10.27275**
**2782**	**0.835896**	**2.072845**	**6.983272**	**0.416894**	**10.30891**
**2805**	**5.378**	**1.04917**	**2.722836**	**1.245206**	**10.39521**
**2243**	**2.416393**	**2.175277**	**2.5773**	**3.479902**	**10.64887**
**2536**	**0.095884**	**3.561788**	**5.82059**	**1.339093**	**10.81736**
**2238**	**0.369387**	**0.742187**	**1.510427**	**8.211061**	**10.83306**
**2750**	**1.707919**	**4.251871**	**4.76343**	**0.150689**	**10.87391**
**2582**	**0.015488**	**7.063076**	**2.857923**	**1.001419**	**10.93791**
**2763**	**0.837641**	**1.910903**	**2.306422**	**6.438629**	**11.4936**
**2712**	**1.220704**	**3.76591**	**0.904302**	**5.723938**	**11.61485**
**2776**	**0.829593**	**6.22405**	**2.933064**	**1.661297**	**11.648**
**2745**	**0.483571**	**7.320622**	**3.363013**	**0.494163**	**11.66137**
**2292**	**6.336444**	**0.755493**	**0.917318**	**3.729713**	**11.73897**
**2313**	**1.667143**	**5.449233**	**1.05964**	**3.572653**	**11.74867**
**2797**	**1.177325**	**2.867035**	**5.626742**	**2.078636**	**11.74974**
**2517**	**1.221803**	**1.909419**	**6.763958**	**1.994974**	**11.89015**
**2772**	**2.646193**	**2.331562**	**4.805164**	**2.226701**	**12.00962**
**2736**	**0.484217**	**1.103899**	**4.32752**	**6.178309**	**12.09394**

2764	0.631487	1.695593	2.203302	7.563906	12.09429
2793	1.489648	1.161479	1.848343	7.710947	12.21042
2795	4.984398	2.095374	3.134766	2.759567	12.9741
2325	1.238588	4.883085	4.696405	2.29939	13.11747
2729	1.588777	0.723542	10.01355	0.941833	13.2677
2309	3.67601	7.875024	1.130204	0.646231	13.32747
2779	4.80094	1.564679	2.307191	4.689823	13.36263
2769	7.138941	0.780596	3.375685	2.178357	13.47358
2780	0	0	0.080836	13.41036	13.4912
2293	2.924648	8.642375	1.481113	0.740947	13.78908
2251	3.64777	6.164063	3.173318	0.833415	13.81857
2741	0.400138	4.647692	7.94182	0.992412	13.98206
2617	4.338716	0.590508	7.669857	1.826844	14.42593
2785	0.430656	3.851002	6.809705	3.641328	14.73269
2669	2.33311	0.755658	3.993671	7.883608	14.96605
2609	14.18666	0.19146	0.534177	0.334837	15.24714
2264	5.988512	1.948305	1.068748	6.533843	15.53941
2324	4.450963	0.542893	10.80094	0.051012	15.84581
2291	11.32019	1.110001	1.255965	2.227192	15.91335
2762	2.502233	8.734668	0.901248	3.885327	16.02348
2751	4.399732	8.68468	0.298078	3.663625	17.04612
2307	7.975978	6.311459	2.093122	0.954098	17.33466
2281	8.308053	1.125215	6.634586	1.30942	17.37727
2276	2.622151	2.280251	5.388041	7.502551	17.79299
2245	0.30772	15.33213	1.894182	1.003903	18.53793
2306	3.757092	6.979365	5.416031	2.409613	18.5621
2787	3.793091	8.192233	2.602365	4.318974	18.90666
2778	7.244116	2.638545	5.773732	4.604549	20.26094

Table S2: Kinase Inhibitor dsRNA Area Under the Curve (AUC)

	Cat#	Item Name	Target	Family Name	dsRNA signal, AU<br>High	Low	Total
1	A8310	PF-562271	Pyk2	TK	0.0	690.0	690.0
2	N1338	Sanguinarine	Metabolic Disease	Other	0.0	895.9	895.9
3	A8619	TIC10	Akt	AGC	0.0	1481.6	1481.6
4	C6184	3-Methylindole	NF-?B	Other	0.0	1602.7	1602.7
5	B1135	GDC-0623	MEK1/2	STE	0.0	1908.1	1908.1
6	B1539	Tideglusib	GSK-3	CMGC	352.7	1603.7	1956.4
7	B1130	GLPG0634	JAK	TK	36.1	2690.2	2726.3
8	N1715	Diosgenin	JAK	TK	0.0	2764.9	2764.9
9	A3628	MLN120B	I?B/IKK	Other	3021.2	0.0	3021.2
10	A3417	Flavopiridol	Cyclin-Dependent Kinases	CMGC	411.3	2633.8	3045.1
11	A3479	Hydroxyfasudil hydrochloride	ROCK	AGC	2331.1	751.7	3082.8
12	A3524	kb NB 142-70	PKD	CAMK	958.0	2847.9	3805.9
13	A4148	NVP-BSK805 2HCl	JAK	TK	1810.2	2038.5	3848.7
14	A4115	JNJ-7706621	Aurora Kinase	Other	0.0	4378.3	4378.3
15	A3535	KX2-391 dihydrochloride	Src	TK	1685.1	2878.5	4563.6
16	A4150	WHI-P154	JAK	TK	5163.1	0.0	5163.1
17	A8628	Chloroquine diphosphate	Autophagy	Other	4180.7	1072.6	5253.3
18	N2206	Mollugin	I?B/IKK	Other	394.8	5157.1	5551.9
19	A3248	BMS345541 hydrochloride	I?B/IKK	Other	0.0	5872.9	5872.9
20	A5566	LY2228820	p38	CMGC	88.7	5927.3	6016.0
21	A8616	A-674563	Akt	AGC	0.0	6156.4	6156.4
22	A5653	AT7867	Akt	AGC	150.0	6554.9	6704.9
23	B1544	Tyrphostin AG 879	HER2	TK	54.0	6760.3	6814.3
24	N2060	12-O-tetradecanoyl phorbol-13-acetate (PMA)	Others	Other	6922.4	0.0	6922.4
25	A8319	Dacomitinib (PF299804, PF299)	EGFR	TK	0.0	7030.9	7030.9
26	N1677	Sennoside B	STAT	TK	3953.3	3310.7	7264.0
27	B5946	EW-7197	TGF-? Receptor	TKL	168.7	7205.0	7373.6
28	B2171	Imatinib (STI571)	c-Kit	TK	4336.5	3075.6	7412.1

29	B4969	KN-93 Phosphate	CaM kinase II	CAMK	4943.4	2840.4	7783.8
30	C4009	CP21R7	GSK-3	CMGC	5189.9	2975.5	8165.4
31	B3252	Dorsomorphin (Compound C)	AMPK	CAMK	6395.6	1963.8	8359.4
32	A8237	SKLB610	VEGFR	TK	126.4	8430.0	8556.4
33	A8301	GW788388	TGF-?R1(ALK5)	TKL	8820.5	0.0	8820.5
34	A3771	RKI-1447	ROCK	AGC	1722.0	7250.9	8972.9
35	A1805	Imatinib Mesylate (STI571)	Bcr-Abl	TK	2759.7	6865.2	9624.9
36	A8253	PD 173074	FGFR	TK	6568.6	3359.6	9928.2
37	A3337	CX-6258	Pim	CAMK	0.0	9957.5	9957.5
38	N2083	Ligustroflavone	I?B/IKK	Other	4049.2	6040.1	10089.3
39	A8618	Palomid 529	Akt	AGC	5778.6	4358.2	10136.8
40	A8412	Dinaciclib (SCH727965)	Cyclin-Dependent Kinases	CMGC	10476.7	0.0	10476.7
41	A8617	CCT128930	Akt	AGC	1507.6	9152.4	10660.0
42	A2080	PIK-75	PI3K	TK	6934.4	3754.5	10688.9
43	A8207	AZD6244 (Selumetinib)	MEK1/2	STE	7299.3	3732.5	11031.8
44	N2179	Cyasterone	DYRK	CMGC	81.6	11248.2	11329.8
45	A4138	Tofacitinib (CP-690550,Tasocitinib)	JAK	TK	6571.2	4801.5	11372.7
46	B2286	K02288	TGF-?R1(ALK5)	TKL	4456.5	7061.1	11517.7
47	N1878	Fumalic acid	Others	Other	4691.7	6874.3	11566.0
48	A5096	PF-04217903	c-MET	TK	9610.7	2049.9	11660.6
49	A8565	Purvalanol B	Cyclin-Dependent Kinases	CMGC	4137.6	7805.6	11943.2
50	A8394	CHIR-124	Chk	CAMK	9794.1	2244.6	12038.6
51	A4193	SMI-4a	Pim	CAMK	0.0	12244.4	12244.4
52	B5817	GDC-0994	MEK1/2	STE	4912.8	7646.0	12558.8
53	A8889	G-749	FLT3	TK	0.0	12828.3	12828.3
54	A8322	Neratinib (HKI-272)	EGFR	TK	1101.0	11881.7	12982.6
55	A3527	Ki20227	c-FMS	TK	0.0	13064.0	13064.0
56	A4139	AG-490	EGFR	TK	8748.3	4415.8	13164.1
57	A3194	AST 487	FLT3	TK	7505.2	5709.8	13214.9
58	B4786	Zotarolimus(ABT-578)	mTOR	Atypical	11435.6	1864.8	13300.4
59	A1986	Nu 6027	Cyclin-Dependent Kinases	CMGC	2596.9	10731.2	13328.1
60	A8249	SB 431542	TGF-?R1(ALK5)	TKL	540.4	12958.4	13498.8
61	A8548	Fingolimod (FTY720)	S1P receptor	Other	0.0	13731.4	13731.4

62	B7947	Aurora Kinase Inhibitor III	Aurora Kinase	Other	2378.0	11511.4	13889.4
63	B4764	TCS-PIM-1-4a	Pim	CAMK	1917.5	12250.9	14168.4
64	A8247	Afatinib (BIBW2992)	EGFR	TK	1924.8	12314.4	14239.2
65	B6054	EAI045	EGFR	TK	0.0	14269.6	14269.6
66	A3530	KN-92 hydrochloride	P2X purinergic receptor	Other	0.0	14359.5	14359.5
67	A4114	MLN8054	Aurora Kinase	Other	4632.4	9929.7	14562.2
68	B4923	TA 01	HSC	Other	672.4	13985.5	14657.9
69	A3576	LY2874455	FGFR	TK	11432.5	3320.6	14753.1
70	A5573	Pimasertib (AS-703026)	MEK1/2	STE	11387.3	3667.2	15054.4
71	A8464	LY2109761	TGF-?R1(ALK5)	TKL	9694.6	5823.5	15518.1
72	B5970	Sanguinarine chloride	Protein Ser/Thr Phosphatases	CMGC	0.0	15548.2	15548.2
73	A4146	CEP-33779	JAK	TK	12244.2	3458.4	15702.6
74	A8223	CID 2011756	Protein Ser/Thr Phosphatases	CMGC	3490.5	12424.4	15914.8
75	A2168	Dovitinib (TKI-258, CHIR-258)	FGFR	TK	15765.0	183.2	15948.2
76	A8312	Torin 1	mTOR	Atypical	1083.7	14896.3	15980.1
77	B1235	NVP 231	CERK	Other	1973.6	14037.2	16010.8
78	B6025	DASA-58	PKM2	Other	7492.3	8577.3	16069.6
79	B2287	LY364947	SMAD	CMGC	12638.5	3456.2	16094.7
80	B7174	DCA	Others	Other	2582.6	13605.1	16187.7
81	A8640	Flavopiridol hydrochloride	Cyclin-Dependent Kinases	CMGC	187.1	16139.6	16326.6
82	A3342	D4476	CK1	CK1	6178.6	10405.5	16584.1
83	A8678	CID 755673	PKD	CAMK	9033.3	7582.1	16615.4
84	B1537	AZD2858	GSK-3	CMGC	10014.5	6684.6	16699.1
85	A8373	AZD2014	mTOR	Atypical	4130.5	12584.4	16714.9
86	B3288	APY29	IRE1	Other	1195.6	15781.0	16976.7
87	A1882	Cediranib (AZD217)	VEGFR	TK	1359.3	15765.2	17124.5
88	B6007	AZD6738	ATM/ATR	Atypical	290.6	16844.7	17135.3
89	A3660	Nilotinib monohydrochloride monohydrate	Bcr-Abl	TK	2759.7	14574.2	17333.9
90	A8689	PD 169316	p38	CMGC	4123.0	13218.6	17341.6
91	B1494	Tyrphostin 9	VEGFR	TK	58.0	17328.1	17386.1
92	B2176	GSK1059615	PI3K	TK	832.8	16639.6	17472.4
93	N1828	Apigenin	Immunology & Inflammation related	Other	1738.8	15810.5	17549.4
94	B3209	Triapine	DNA Synthesis	Other	0.0	17637.1	17637.1

95	A2754	TG100-115	PI3K	TK	401.6	17405.4	17807.0
96	A3931	VX-11e	ERK	CMGC	6059.4	11825.4	17884.8
97	A3197	AT7519 Hydrochloride	Cyclin-Dependent Kinases	CMGC	0.0	18031.2	18031.2
98	A4136	TG101348 (SAR302503)	JAK	TK	3065.1	15118.2	18183.4
99	B3275	TCS JNK 5a	JNK	CMGC	1144.9	17159.4	18304.3
100	B1124	Pitavastatin	HMG-CoA Reductase	Other	8150.8	10215.5	18366.3
101	A4521	TCS PIM-1 1	Pim	CAMK	1935.2	16463.0	18398.2
102	A8350	AZD4547	FGFR	TK	14827.8	3634.0	18461.7
103	A1404	Rigosertib (ON-01910,Estybon)	PLK	Other	14175.0	4849.9	19024.8
104	B7808	NT157	IGF1R	TK	7372.8	11852.6	19225.3
105	B2173	CP-673451	VEGFR	TK	14720.6	4566.7	19287.4
106	N1908	Ginkgolide C	P450 (e.g. CYP17)	TK	4330.9	14963.6	19294.5
107	B1969	Mesalamine	PGE synthase	AGC	17187.6	2129.6	19317.3
108	A3861	TBB	CK2	CMGC	3323.3	16520.0	19843.3
109	A3719	PF-670462	CK1	CK1	1350.7	18716.0	20066.6
110	A8638	LY2603618	Chk	CAMK	13995.2	6197.3	20192.5
111	A4182	Resveratrol	Sirtuin	Other	13503.5	6902.6	20406.1
112	A3389	EMD638683	SGK	AGC	288.1	20264.2	20552.3
113	B1372	Dorsomorphin 2HCl	AMPK	CAMK	1809.4	18817.3	20626.7
114	A5707	PD318088	MEK1/2	STE	9157.7	11553.5	20711.2
115	A3532	KN-93	CaM kinase II	CAMK	14156.8	6603.8	20760.7
116	A2278	NVP-AEW541	IGF1R	TK	1274.0	19640.6	20914.5
117	A8407	Dibucaine (Cinchocaine) HCl	Sodium Channel	Other	9319.1	11778.5	21097.5
118	B5815	LY2584702	S6 Kinase	AGC	5638.3	15579.4	21217.6
119	B1970	Metformin HCl	Others	Other	10385.6	10840.1	21225.7
120	A3001	PCI-32765 (Ibrutinib)	BTK	TK	3646.9	17695.2	21342.1
121	A3751	Regorafenib monohydrate	c-RET	TK	7085.4	14285.8	21371.2
122	A3531	KN-92 phosphate	P2X purinergic receptor	Other	4429.3	16946.8	21376.0
123	A5979	Tie2 kinase inhibitor	Tie-2	TK	4787.9	16594.7	21382.6
124	A8476	MK-2461	PDGFR	TK	19211.6	2226.3	21437.9
125	A5639	BIRB 796 (Doramapimod)	p38	CMGC	8315.3	13140.6	21455.9
126	A8226	TAK-632	Raf	TKL	12667.4	8796.8	21464.2
127	A2477	Tyrphostin AG 1296	PDGFR	TK	2647.7	18881.3	21528.9
128	A5793	Quizartinib (AC220)	FLT3	TK	4499.9	17036.5	21536.5

129	A3940	XL388	mTOR	Atypical	5203.5	16443.4	21646.9
130	A3148	AIM-100	Ack1	TK	17272.0	4414.3	21686.3
131	C6104	L-Leucine	Anti-infection	Other	3716.4	18120.1	21836.5
132	A3184	AR-A014418	GSK-3	CMGC	79.4	22152.5	22232.0
133	A3397	Erlotinib	EGFR	TK	7272.0	15317.7	22589.7
134	B6674	Anisomycin	JNK	CMGC	20082.7	2629.3	22712.1
135	A3750	Regorafenib hydrochloride	c-RET	TK	724.9	22021.0	22745.9
136	B5663	SC 79	Akt	AGC	17618.8	5138.7	22757.5
137	A3125	5-Iodotubercidin	Adenosine Kinase	Other	2639.0	20167.4	22806.4
138	A4213	Necrostatin-1	TNF-?	CMGC	12590.5	10276.4	22866.9
139	A4510	1,2,3,4,5,6-Hexabromocyclohexane	JAK	TK	58.9	23091.7	23150.6
140	A3321	Cobimetinib	MEK1/2	STE	1412.8	21814.8	23227.7
141	A8304	FR 180204	ERK	CMGC	10806.0	12597.7	23403.7
142	A8347	Pazopanib Hydrochloride	VEGFR	TK	14157.8	9479.9	23637.7
143	A3570	LY2090314	GSK-3	CMGC	16169.3	7523.7	23693.1
144	B1587	IMD 0354	I?B/IKK	Other	3341.9	20862.1	24204.0
145	A4151	ZM 39923 HCl	JAK	TK	21395.1	2858.2	24253.3
146	B1401	Cabozantinib malate (XL184)	c-MET	TK	148.7	24276.1	24424.8
147	B5942	AS601245	Others	Other	18398.2	6237.2	24635.4
148	A2198	Genistein	Topoisomerase	Other	5526.1	19461.3	24987.4
149	A8883	SAR405	Autophagy	Other	1587.9	23454.9	25042.7
150	N2131	Wedelolactone	EGFR	TK	9001.4	16105.5	25106.9
151	B2276	Vinpocetine	PDE	Other	5212.2	19993.1	25205.3
152	A4333	CPI-613	Dehydrogenase	Other	36.8	25399.4	25436.2
153	B5712	ANA 12	Trk	TK	5083.5	20498.0	25581.6
154	A1952	NSC 23766	Rho	AGC	10192.1	15408.3	25600.4
155	A1723	Roscovitine (Seliciclib,CYC202)	Cyclin-Dependent Kinases	CMGC	8144.1	17609.2	25753.3
156	A8890	HTH-01-015	AMPK	CAMK	4731.3	21129.9	25861.2
157	A8303	GNF-5837	Trk	TK	2750.0	23319.5	26069.5
158	A5331	TSU-68 (SU6668,Orantinib)	VEGFR	TK	4841.4	21696.8	26538.2
159	B1642	WYE-125132 (WYE-132)	mTOR	Atypical	4894.7	21738.1	26632.8
160	A3504	IRAK inhibitor 6	IRAK	TKL	19776.5	7334.0	27110.5
161	A1450	Lidocaine	Histamine Receptor	Other	26152.6	999.3	27151.9
162	A3347	Dabrafenib Mesylate (GSK-2118436)	Raf	TKL	27414.0	0.0	27414.0

163	B8365	LY3214996	ERK	CMGC	4594.5	22927.9	27522.4
164	A2949	Linifanib (ABT-869)	VEGFR	TK	6252.9	21480.2	27733.1
165	A8885	Ro 3306	Cyclin-Dependent Kinases	CMGC	2807.1	25048.4	27855.5
166	A3794	SB1317	JAK	TK	1210.9	26680.2	27891.1
167	A3433	Gefitinib hydrochloride	EGFR	TK	0.0	28000.5	28000.5
168	A2306	Ellagic acid	Topoisomerase	Other	14274.7	14007.9	28282.5
169	A8636	RN486	BTK	TK	3432.0	25024.9	28456.9
170	B6116	GLPG0634-A	JAK	TK	20519.6	8028.8	28548.5
171	A3006	GDC-0068 (RG7440)	Akt	AGC	10110.1	18551.4	28661.5
172	B4877	URMC-099	Others	Other	20784.2	7905.7	28690.0
173	B3697	4E1RCat	Others	Other	11534.6	17206.1	28740.7
174	A8248	ZSTK474	PI3K	TK	6069.9	23073.5	29143.5
175	A5506	Thiazovivin	ROCK	AGC	2364.1	26827.3	29191.4
176	B2186	GSK2636771	PI3K	TK	841.3	28354.9	29196.1
177	C4445	2-D08	SUMOylation	Other	21678.5	7689.9	29368.4
178	A3206	AVL-292	BTK	TK	20034.7	9433.9	29468.6
179	B1621	TAK-733	MEK1/2	STE	15107.2	14755.9	29863.1
180	B1585	SC-514	I?B/IKK	Other	620.3	29713.1	30333.4
181	A8199	PD153035 hydrochloride	EGFR	TK	1669.4	28866.3	30535.6
182	A3943	XMD8-92	ERK	CMGC	359.5	30524.1	30883.6
183	A2412	CP-724714	EGFR	TK	1118.3	29983.8	31102.1
184	B1409	Benidipine HCl	Calcium Channel	Other	130.4	31078.1	31208.6
185	N1802	Oridonin	Anti-infection	Other	6061.2	25375.1	31436.3
186	A1655	GW2580	CSF-1R	TK	17974.9	13531.9	31506.8
187	A4192	SGI-1776 free base	Pim	CAMK	1583.1	29976.5	31559.5
188	A2552	Quercetin dihydrate	PI3K	TK	25411.6	6435.1	31846.8
189	A8551	INK 128 (MLN0128)	mTOR	Atypical	0.0	31875.7	31875.7
190	B3702	MSDC-0160	Others	Other	16528.8	15455.5	31984.3
191	A3392	Emodin	NF-?B	Other	20341.5	11688.8	32030.2
192	B2182	CAY10505	PI3K	TK	15375.8	16784.8	32160.6
193	A3792	SB 239063	p38	CMGC	16458.2	15855.6	32313.9
194	B4492	HS-173	PI3K	TK	345.7	31987.4	32333.1
195	B1525	SSR128129E	FGFR	TK	4736.0	27624.5	32360.5
196	A3306	Chelerythrine Chloride	PKC	AGC	10306.1	22120.5	32426.7
197	B5937	KPT-9274	PAK4	STE	6803.0	25813.6	32616.6
198	N1849	Dihydromyricetin	MEK	STE	20863.8	12380.4	33244.2
199	B1011	Bafetinib (INNO-406)	Bcr-Abl	TK	1113.6	32473.2	33586.8

**200**	**B4842**	**LDN-214117**	**ALK**	**TK**	**4693.4**	**28899.6**	**33593.0**
**201**	**B1249**	**TDZD-8**	**GSK-3**	**CMGC**	**7580.3**	**26092.4**	**33672.7**
**202**	**B8316**	**Harmine**	**PPAR,MAO**	**Other**	**9958.7**	**24294.0**	**34252.7**
**203**	**A8902**	**6H05**	**Rho**	**AGC**	**9210.5**	**25435.6**	**34646.0**
**204**	**B1405**	**GW5074**	**Raf**	**TKL**	**14683.4**	**20240.6**	**34924.0**
**205**	**A3222**	**Baricitinib phosphate**	**JAK**	**TK**	**2518.9**	**32442.1**	**34961.0**
**206**	**B3570**	**CGP 57380**	**Others**	**Other**	**1947.6**	**33084.2**	**35031.8**
**207**	**A4152**	**BMS-911543**	**JAK**	**TK**	**18362.9**	**16684.7**	**35047.6**
**208**	**A1894**	**SL-327**	**MEK1/2**	**STE**	**10911.9**	**24198.7**	**35110.7**
**209**	**A3887**	**Trametinib DMSO solvate**	**MEK1/2**	**STE**	**10918.5**	**24279.8**	**35198.3**
**210**	**A3448**	**GSK2606414**	**PERK**	**Other**	**6871.8**	**28375.6**	**35247.4**
**211**	**A2846**	**OSU-03012 (AR-12)**	**PDK-1**	**AGC**	**17565.3**	**17813.3**	**35378.6**
**212**	**B2175**	**GSK2656157**	**PERK**	**Other**	**2371.9**	**33522.6**	**35894.5**
**213**	**A8546**	**R406**	**Spleen Tyrosine Kinase (Syk)**	**TK**	**5357.1**	**30563.4**	**35920.5**
**214**	**B1496**	**Icotinib**	**EGFR**	**TK**	**11365.1**	**24561.1**	**35926.2**
**215**	**B4809**	**K-115**	**Rho**	**AGC**	**25318.1**	**10922.3**	**36240.4**
**216**	**A3923**	**VO-Ohpic trihydrate**	**PTEN**	**TK**	**28838.2**	**7631.3**	**36469.5**
**217**	**A8320**	**PF-00562271**	**FAK**	**TK**	**0.0**	**36485.5**	**36485.5**
**218**	**N1308**	**Biochanin A**	**Antioxidant**	**Other**	**14982.3**	**21751.5**	**36733.9**
**219**	**B2303**	**Apatinib**	**VEGFR**	**TK**	**181.1**	**36614.7**	**36795.8**
**220**	**B4986**	**LY2409881**	**I?B/IKK**	**Other**	**22360.9**	**15064.3**	**37425.3**
**221**	**A8324**	**LDN-193189**	**SMAD**	**CMGC**	**30043.0**	**7399.9**	**37442.8**
**222**	**A4120**	**MK-5108 (VX-689)**	**Aurora Kinase**	**Other**	**3258.6**	**34308.8**	**37567.4**
**223**	**B4904**	**ACTB-1003**	**FGFR**	**TK**	**28009.9**	**9584.0**	**37593.9**
**224**	**A3626**	**ML-7 hydrochloride**	**ATPase**	**Other**	**19324.6**	**18346.9**	**37671.5**
**225**	**N1827**	**Artemisinine**	**P450 (e.g. CYP17)**	**TK**	**2826.9**	**35035.3**	**37862.2**
**226**	**A3529**	**KN-92**	**CaM kinase II**	**CAMK**	**8269.0**	**30018.5**	**38287.5**
**227**	**B6115**	**RG 13022**	**EGFR**	**TK**	**36154.5**	**2414.3**	**38568.8**
**228**	**A2600**	**(-)-Epigallocatechin gallate (EGCG)**	**PKC**	**AGC**	**14230.9**	**24448.0**	**38678.9**
**229**	**A1632**	**SB202190 (FHPI)**	**p38**	**CMGC**	**29217.1**	**9508.1**	**38725.2**
**230**	**B9000**	**8-Bromo-cAMP, sodium salt**	**cAMP**	**AGC**	**25859.7**	**12957.0**	**38816.6**
**231**	**A4237**	**Amuvatinib (MP-470, HPK 56)**	**c-RET**	**TK**	**6649.7**	**32179.3**	**38829.0**
**232**	**A4121**	**SNS-314 Mesylate**	**Aurora Kinase**	**Other**	**5716.5**	**33317.4**	**39033.9**
**233**	**A3805**	**SCH772984**	**MEK1/2**	**STE**	**21169.8**	**17905.7**	**39075.6**
**234**	**A8504**	**Pitavastatin Calcium**	**HMG-CoA Reductase**	**Other**	**16024.1**	**23195.0**	**39219.2**
**235**	**A8881**	**WZ3146**	**EGFR**	**TK**	**23148.6**	**16337.5**	**39486.1**

236	B5487	EHT 1864	Amyloid ?	Other	22471.9	17099.7	39571.7
237	A8214	AZD8055	mTOR	Atypical	0.0	39910.8	39910.8
238	A3193	ASP3026	ALK	TK	11916.3	27995.4	39911.7
239	A8318	PP242	mTOR	Atypical	7321.2	32614.7	39936.0
240	B5853	MHY1485	mTOR	Atypical	4962.4	35655.5	40617.9
241	B6193	AMG 337	c-MET	TK	6788.1	33845.4	40633.5
242	B5950	AZD8186	PI3K	TK	4769.3	35908.0	40677.3
243	A2067	PI-103	PI3K	TK	0.0	40731.1	40731.1
244	A1792	PD184352 (CI-1040)	MEK1/2	STE	0.0	41067.4	41067.4
245	B5846	Radotinib(IY-5511)	Bcl-2 Family	TK	11726.8	29413.0	41139.9
246	C4953	HA-100 (hydrochloride)	Broad Spectrum Protein Kinase Inhibitor	Other	27295.9	14084.7	41380.5
247	A3676	NVP-LCQ195	Cyclin-Dependent Kinases	CMGC	6040.0	35350.1	41390.1
248	N1743	Curcumol	Others	Other	0.0	41441.3	41441.3
249	B4907	Mps1-IN-1	Mps1	Other	7290.6	34349.9	41640.6
250	A5092	JNJ-38877605	c-MET	TK	9345.0	32468.4	41813.4
251	A3556	LKB1 (AAK1 dual inhibitor)	Pim	CAMK	42067.1	0.0	42067.1
252	A8336	KU-60019	ATM/ATR	Atypical	31471.9	10671.9	42143.8
253	B5832	Altiratinib	c-MET	TK	7645.1	34585.3	42230.4
254	A4110	MLN8237 (Alisertib)	Aurora Kinase	Other	9976.5	32491.8	42468.3
255	B1536	AZD1080	GSK-3	CMGC	30210.5	12322.5	42533.0
256	B4357	UNC2881	Axl	TK	446.4	42094.2	42540.5
257	A3320	CO-1686 (AVL-301)	EGFR	TK	16434.5	26200.1	42634.6
258	B4787	SF1670	PTEN	TK	30745.4	12301.2	43046.5
259	A8326	AZD-5438	Cyclin-Dependent Kinases	CMGC	10643.7	32871.5	43515.3
260	C5579	Brilliant Blue G	Others	Other	11949.6	31753.4	43703.1
261	A3505	IRAK-1-4 Inhibitor I	IRAK	TKL	21347.7	22363.0	43710.7
262	A1173	AG-18	EGFR	TK	29672.3	14434.9	44107.2
263	A8711	DDD107498	Antimalaria	Other	38942.1	5409.3	44351.3
264	B4094	NMS-1286937	PLK	Other	5848.8	38547.7	44396.5
265	A5611	GSK429286A	ROCK	AGC	10329.9	34075.4	44405.3
266	A1196	SGX-523	c-MET	TK	18702.9	25732.4	44435.3
267	B2219	EHop-016	Rho	AGC	0.0	44568.8	44568.8
268	B4660	PI-3065	PI3K	TK	625.6	44145.1	44770.7
269	B3696	4EGI-1	Others	Other	27081.3	17726.3	44807.6

270	B1760	Flufenamic acid	AMPK; Calcium Channel; Chloride Channel; COX; Potassium Channel	CAMK	29762.4	15182.6	44945.1
271	B2032	SRPIN340	SRPK	CMGC	38969.9	6071.8	45041.7
272	A8184	AICAR	AMPK	CAMK	10649.1	34470.9	45119.9
273	A3209	AXL1717	IGF1R	TK	13380.1	31807.4	45187.5
274	A5071	GDC-0879	Raf	TKL	7041.8	38492.2	45534.0
275	A5072	GSK690693	Akt	AGC	6007.3	39582.1	45589.3
276	A8251	TAE684 (NVP-TAE684)	ALK	TK	41419.4	4182.3	45601.7
277	A8679	CRT 0066101	PKD	CAMK	0.0	45629.7	45629.7
278	B8328	AZD1390	ATM/ATR	Atypical	7959.8	37767.2	45727.1
279	A1169	10058-F4	c-Myc	Other	21785.0	24031.2	45816.2
280	A2673	AG-1024	IGF1R	TK	1211.9	44737.5	45949.4
281	N1768	Rosmarinic acid	Others	Other	3864.4	42457.9	46322.3
282	A2307	PHA-665752	c-MET	TK	34815.2	11571.8	46387.0
283	B1236	BML-277	Chk	CAMK	33021.5	13488.9	46510.4
284	C5108	Amlexanox	Melatonin Receptors	Other	17956.6	28672.3	46628.9
285	A4132	CCT137690	Aurora Kinase	Other	0.0	46657.6	46657.6
286	A5057	MGCD-265	Tie-2	TK	4249.4	42710.5	46959.9
287	A3018	Trametinib (GSK1120212)	MEK1/2	STE	36456.5	10541.3	46997.8
288	A3014	BGJ398	FGFR	TK	24356.1	22673.4	47029.5
289	B1262	HG-10-102-01	LRRK2	TKL	14367.4	32713.5	47080.9
290	B7912	Pyridoxine	Vitamin	Other	17847.0	29400.2	47247.2
291	B6017	AZD3264	I?B/IKK	Other	223.2	47155.4	47378.5
292	A8625	CP-466722	ATM/ATR	Atypical	23326.1	24439.1	47765.2
293	A8525	Sotrastaurin (AEB071)	PKC	AGC	6965.2	40818.2	47783.4
294	A8348	LY2157299	TGF-?R1(ALK5)	TKL	33079.0	15107.1	48186.1
295	B1492	PYR-41	E1 Activating	Other	41809.3	6659.6	48468.9
296	A3353	DB07268	JNK	CMGC	10652.5	37894.5	48547.0
297	B6171	CC-223	mTOR	Atypical	6587.4	41988.9	48576.2
298	B5484	7,8-Dihydroxyflavone	Trk	TK	9230.6	39399.4	48630.0
299	A3354	DCC-2618	PDGFR	TK	31390.7	17341.0	48731.7
300	B6114	CC-115	DNA-PK	Other	5666.1	44138.8	49805.0
301	B1293	Y-27632	ROCK	AGC	3508.5	46665.7	50174.2
302	A8620	AZD-3463	ALK	TK	9390.1	40804.7	50194.8

303	A8541	Triciribine	Akt	AGC	3840.6	46439.7	50280.3
304	A3420	FMK	S6 Kinase	AGC	36246.0	14082.6	50328.7
305	B4688	3CAI	Akt	AGC	6736.4	43810.0	50546.4
306	B2189	YM201636	PIKfyve	TK	7268.3	43298.6	50566.9
307	A8300	ZCL278	Cdc42	CMGC	9541.3	41590.0	51131.3
308	B2297	GW441756	Trk	TK	33214.4	18067.5	51281.9
309	A1387	AZD5363	Akt	AGC	11890.0	39804.7	51694.7
310	A2597	Brivanib (BMS-540215)	VEGFR	TK	51042.4	684.7	51727.0
311	B4754	LDC000067	Cyclin-Dependent Kinases	CMGC	31035.0	20712.1	51747.0
312	A4141	Baricitinib (LY3009104, INCB028050)	JAK	TK	27614.1	24279.2	51893.3
313	A2977	Cabozantinib (XL184, BMS-907351)	c-MET	TK	13075.8	39205.8	52281.6
314	B4990	Purvalanol A	Cyclin-Dependent Kinases	CMGC	51443.1	865.0	52308.0
315	A2689	Butein	EGFR	TK	38311.3	14009.4	52320.7
316	A4143	CYT387	JAK	TK	36485.3	15837.3	52322.6
317	B1162	FRAX597	PAK1	STE	1538.8	50796.9	52335.7
318	A3302	CGI-1746	BTK	TK	34116.6	18368.1	52484.7
319	B2226	SKI II	S1P receptor	Other	31334.0	21341.0	52674.9
320	B4924	TA 02	HSC	Other	8658.0	44038.4	52696.4
321	A3013	PD0325901	MEK1/2	STE	5022.8	48059.9	53082.7
322	C5386	N-Acetylserotonin	MKK	CMGC	219.3	53222.8	53442.1
323	B6108	IC261	CK1	CK1	6564.9	46952.8	53517.7
324	A8354	A66	PI3K	TK	12407.5	41352.4	53759.9
325	A8661	MNS	Integrin	Other	1958.5	51870.8	53829.2
326	N2182	Harmine hydrochloride	STAT3	TK	0.0	53937.1	53937.1
327	A8240	SB 216763	GSK-3	CMGC	596.7	53506.5	54103.1
328	C5638	13(Z)-Docosenoic Acid	Others	Other	4336.2	49926.0	54262.2
329	A5112	XL147	PI3K	TK	6847.8	47539.1	54386.9
330	B1639	Ridaforolimus (Deforolimus, MK-8669)	mTOR	Atypical	23072.2	31512.5	54584.8
331	N1753	Scutellarin	I?B/IKK	Other	1769.9	52840.4	54610.3
332	A8250	LY 294002	PI3K	TK	11164.3	43579.3	54743.6
333	A3145	Afatinib dimaleate	HER2	TK	15371.8	40279.2	55651.0
334	B7853	Tandutinib (MLN518) HCl	FLT3	TK	42444.4	13615.8	56060.2
335	A8234	Erlotinib Hydrochloride	EGFR	TK	3624.7	52457.7	56082.4

336	A3432	GDC-0941 dimethanesulfonate	PI3K	TK	20950.7	35251.8	56202.5
337	A3674	NVP-BKM120 Hydrochloride	PI3K	TK	0.0	56514.5	56514.5
338	A8330	CX-4945 (Silmitasertib)	CK2	CMGC	35326.5	21811.4	57137.9
339	A4488	Anacardic acid	Aurora Kinase	Other	12779.5	44703.4	57482.9
340	A3939	XL228	Aurora Kinase	Other	17836.6	39819.2	57655.8
341	A8210	GDC-0941	PI3K	TK	590.8	57346.3	57937.2
342	B3688	ML347	TGF-?R1(ALK5)	TKL	0.0	58224.3	58224.3
343	A5703	BMS-777607	c-MET	TK	39539.6	18689.6	58229.2
344	N2643	Shikonin		Other	54262.1	4009.3	58271.4
345	A8308	PH-797804	p38	CMGC	733.9	57938.0	58671.9
346	B3686	DMH-1	TGF-?R1(ALK5)	TKL	13545.0	45359.2	58904.2
347	A8353	3-Methyladenine	Autophagy	Other	32357.3	26977.2	59334.5
348	N1620	Ginsenoside Rb1	Others	Other	7414.4	52585.8	60000.1
349	A3847	SU5416	c-RET	TK	39551.1	20455.1	60006.1
350	B1439	Golvatinib (E7050)	c-MET	TK	36777.1	23267.5	60044.6
351	N2031	Piceatannol	PKC; SPHK	AGC	12025.1	48262.2	60287.2
352	A8167	Rapamycin (Sirolimus)	mTOR	Atypical	1745.1	58744.6	60489.7
353	A2065	IC-87114	PI3K	TK	0.0	60540.6	60540.6
354	C6276	Ethyl gallate	Others	Other	180.5	60976.1	61156.7
355	A5176	AS-605240	PI3K	TK	11785.5	49405.3	61190.8
356	A1663	PD98059	MEK1/2	STE	33353.3	27955.5	61308.8
357	A3005	CAL-101 (Idelalisib, GS-1101)	PI3K	TK	57616.6	3708.3	61324.9
358	A8489	NVP-BVU972	c-MET	TK	9035.4	52705.1	61740.5
359	A3388	EMD-1214063	c-MET	TK	14120.6	47689.1	61809.6
360	B3661	(+)-Usniacin	Others	Other	3049.6	59009.6	62059.2
361	N1958	Galangin;3,5,7-Trihydroxyflavone	I?B/IKK	Other	16189.5	46015.9	62205.4
362	A3149	AKT inhibitor VIII	Akt	AGC	14379.0	47838.1	62217.1
363	A8232	Nilotinib(AMN-107)	Bcr-Abl	TK	28653.4	33622.0	62275.3
364	A3732	Poloxin	PLK	Other	3268.7	59035.4	62304.1
365	B7716	Senexin A	CDK	CMGC	30520.6	31796.6	62317.2
366	B1431	TG003	CDK	CMGC	1126.7	61445.9	62572.6
367	A5602	SB525334	TGF-?R1(ALK5)	TKL	34680.3	27941.4	62621.6
368	A1821	Ki8751	VEGFR	TK	16810.1	45822.4	62632.5
369	A2822	AC480 (BMS-599626)	EGFR	TK	468.1	62401.2	62869.3
370	A1186	AMG-208	c-MET	TK	40405.8	22501.3	62907.1
371	A8603	GNF 2	Bcr-Abl	TK	2578.1	60765.8	63344.0

372	A8236	Regorafenib	c-RET	TK	93.0	63322.3	63415.2
373	A5803	BIX 02188	MEK1/2	STE	40230.6	23376.7	63607.2
374	B1371	Miltefosine	Akt	AGC	27276.6	36330.6	63607.3
375	A3022	Pazopanib (GW-786034)	PDGFR	TK	41143.8	22671.3	63815.1
376	B2190	H 89 2HCl	PKA	AGC	7807.6	56279.7	64087.4
377	A8418	Dovitinib Dilactic acid	VEGFR	TK	47107.5	17331.2	64438.8
378	A3260	BMX-IN-1	BMX Kinase	TK	37737.5	26747.2	64484.7
379	A8329	R428	Axl	TK	1050.4	64667.0	65717.4
380	A8325	Tivantinib (ARQ 197)	c-MET	TK	46631.5	19485.6	66117.2
381	A8604	GNF 5	Bcr-Abl	TK	498.5	65630.6	66129.1
382	A8550	Telatinib (BAY 57-9352)	VEGFR	TK	46565.4	19646.6	66212.0
383	B5860	TAK960	PLK	Other	9929.6	56355.2	66284.8
384	B9001	Dibutyryl-cAMP, sodium salt	cAMP	AGC	10702.1	55803.3	66505.4
385	A3545	LDN193189 Hydrochloride	SMAD	CMGC	42525.8	24650.0	67175.8
386	B1998	Oxfendazole	Anti-infection	Other	20012.9	47370.5	67383.3
387	B3699	ISRIB (trans-isomer)	PERK	Other	6980.3	60689.2	67669.5
388	B1543	Mubritinib (TAK 165)	HER2	TK	19342.6	48533.2	67875.8
389	B1027	2-Deoxy-D-glucose	Hexokinase	Other	5572.7	62378.7	67951.4
390	B1641	WAY-600	mTOR	Atypical	1003.4	67106.4	68109.8
391	A8357	AG-1478	EGFR	TK	1676.2	67129.9	68806.1
392	B1374	WZ4003	AMPK	CAMK	30589.4	38446.0	69035.5
393	A2323	TCS 359	FLT3	TK	5896.0	63380.8	69276.8
394	A5719	AT7519	Cyclin-Dependent Kinases	CMGC	23488.0	46607.1	70095.0
395	B1437	PF-477736	Chk	CAMK	45938.3	24364.8	70303.1
396	B7850	BMS-582949 hydrochloride	p38	CMGC	7441.1	64364.9	71806.0
397	A8688	TAK-715	p38	CMGC	42569.8	29513.1	72082.8
398	B2114	Mitoxantrone HCl	Topoisomerase	Other	48235.0	23848.1	72083.1
399	N1609	Ginsenoside Rh2	Others	Other	41827.1	30569.1	72396.2
400	A3011	CHIR-99021 (CT99021)	GSK-3	CMGC	62333.9	10204.7	72538.6
401	B7815	NSC228155	EGFR	TK	18547.9	54998.4	73546.3
402	N1789	Salidroside	Others	Other	19295.0	56220.3	75515.3
403	N1615	Ginsenoside Re	Others	Other	10054.1	65920.2	75974.2
404	A2251	Tivozanib (AV-951)	VEGFR	TK	40142.9	36133.0	76275.9
405	A3760	Reversine	Aurora Kinase	Other	27234.6	49415.3	76649.9
406	B5816	PLX647	c-FMS	TK	24285.0	52941.2	77226.3

407	B1540	TWS119	GSK-3	CMGC	48959.2	29417.1	78376.3
408	B2301	SAR131675	VEGFR	TK	44218.5	34484.3	78702.8
409	A8343	Go 6983	PKC	AGC	52923.0	26190.1	79113.1
410	A4512	Cucurbitacin I	JAK	TK	39627.9	39750.1	79378.0
411	A2149	Bosutinib (SKI-606)	Bcr-Abl	TK	38090.7	42027.4	80118.1
412	A3825	SLx-2119	ROCK	AGC	20785.5	59494.3	80279.8
413	B8023	Cerdulatinib (PRT062070)	JAK	TK	60131.0	21245.1	81376.1
414	B3300	Cromolyn sodium	Calcium Channel	Other	64024.7	17957.1	81981.8
415	A2174	Lenvatinib (E7080)	VEGFR	TK	26964.0	55504.0	82468.0
416	B4761	GNE-9605	LRRK2	TKL	40803.1	41939.9	82743.0
417	B5827	Poziotinib	HER2	TK	19439.7	63934.7	83374.5
418	A8215	PP 1	Src	TK	19934.9	63796.3	83731.2
419	A3132	A 77-01	TGF-?R1(ALK5)	TKL	63808.0	20371.9	84180.0
420	B3553	GS-9973	Spleen Tyrosine Kinase (Syk)	TK	32878.5	51541.3	84419.8
421	B4898	Bikinin	GSK-3	CMGC	39833.6	44813.6	84647.2
422	C4882	1,2-Dilauroyl-sn-glycerol	PKC	AGC	32229.3	52450.9	84680.2
423	B3033	Bay 11-7085	I?B/IKK	Other	63723.6	21705.2	85428.7
424	N1345	Obacunone	Others	Other	22534.9	63087.7	85622.6
425	A3575	LY2835219 free base	Cyclin-Dependent Kinases	CMGC	59180.8	26611.9	85792.7
426	A4605	KU 55933	ATM/ATR	Atypical	33867.0	52307.6	86174.6
427	A1947	MEK162 (ARRY-162, ARRY-438162)	MEK1/2	STE	32104.0	54835.5	86939.5
428	B4800	Defactinib	FAK	TK	31683.7	56452.3	88136.0
429	A4112	Barasertib (AZD1152-HQPA)	Aurora Kinase	Other	48647.4	41038.0	89685.3
430	A5760	KRN 633	VEGFR	TK	55097.1	35164.2	90261.3
431	A8374	AZD8330	MEK1/2	STE	29149.3	63631.5	92780.8
432	A3965	BI 2536	PLK	Other	34739.4	58236.8	92976.2
433	C5545	MK2 Inhibitor IV	P2X purinergic receptor	Other	42817.4	50349.5	93166.9
434	B4906	PF-06447475	LRRK2	TKL	53174.2	40604.3	93778.5
435	A8216	PP 2 (AG 1879)	Src	TK	53589.5	40414.9	94004.3
436	A3717	PF-543	S1P receptor	Other	28127.6	66805.4	94933.0
437	B1402	GZD824	Bcr-Abl	TK	64275.0	33439.8	97714.8
438	A5880	R406 (free base)	Spleen Tyrosine Kinase (Syk)	TK	42129.8	55638.2	97768.1
439	A8395	CHIR-98014	GSK-3	CMGC	63043.9	37241.6	100285.6
440	B5624	STF 083010	AChE	Other	54363.2	46405.9	100769.1

**441**	**B5952**	**LFM-A13**	**BTK**	**TK**	**60025.3**	**42758.8**	**102784.1**
**442**	**A4124**	**TAK-901**	**Aurora Kinase**	**Other**	**42486.6**	**61332.6**	**103819.2**
**443**	**B5854**	**Pexidartinib (PLX3397)**	**CSF-1R**	**TK**	**64762.3**	**40586.0**	**105348.3**
**444**	**A8370**	**Axitinib (AG 013736)**	**VEGFR**	**TK**	**42600.6**	**65444.2**	**108044.8**
**445**	**A3519**	**JNK-IN-7**	**JNK**	**CMGC**	**66910.1**	**45982.8**	**112893.0**
**446**	**B1974**	**Methylthiouracil**	**Others**	**Other**	**61524.3**	**55636.1**	**117160.4**
**447**	**A4135**	**Tofacitinib (CP-690550) Citrate**	**JAK**	**TK**	**57107.2**	**61115.5**	**118222.7**
**448**	**A4541**	**Sal 003**	**Protein Ser/Thr Phosphatases**	**CMGC**	**58234.6**	**61292.8**	**119527.4**
**449**	**A8448**	**INCB28060**	**c-MET**	**TK**	**59276.3**	**63091.3**	**122367.6**
**450**	**B8016**	**UNC2025**	**FLT3**	**TK**	**55553.7**	**67297.4**	**122851.1**
